# Connecting Actors With the Introduction of Mobile Technology in Health Care Practice Placements (4D Project): Protocol for a Mixed Methods Study

**DOI:** 10.2196/53284

**Published:** 2024-02-08

**Authors:** Carlos Martínez-Gaitero, Sebastian Maximilian Dennerlein, Beata Dobrowolska, Angela Fessl, Daniel Moreno-Martínez, Stephanie Herbstreit, Gilbert Peffer, Esther Cabrera, 4D Project Consortium

**Affiliations:** 1 Tecnocampus Pompeu Fabra University Mataró Spain; 2 Faculty of Behavioural, Management and Social Sciences University of Twente Enschede Netherlands; 3 Faculty of Health Sciences Medical University of Lublin Lublin Poland; 4 Institute of Interactive Systems and Data Science Graz University of Technology Graz Austria; 5 Know-Center GmbH Graz Austria; 6 Germans Trias i Pujol Research Institute Hospital Germans Trias i Pujol Institut Català de la Salut Badalona Spain; 7 Medical Faculty University of Duisburg-Essen Essen Germany; 8 Kubify Utrecht Netherlands; 9 see Acknowledgments

**Keywords:** practice-based learning, practice placement, technology enhanced learning, mobile learning, co-design, cocreation, higher education, health professionals, health students.

## Abstract

**Background:**

The learning process in clinical placements for health care students is a multifaceted endeavor that engages numerous actors and stakeholders, including students, clinical tutors, link teachers, and academic assessors. Successfully navigating this complex process requires the implementation of tasks and mentorships that are synchronized with educational and clinical processes, seamlessly embedded within their respective contexts. Given the escalating number of students and the rising demand for health care services from the general population, it becomes imperative to develop additional tools that support the learning process. These tools aim to simplify day-to-day clinical practice, allowing a concentrated focus on value-based activities. This paper introduces a project funded by the European Commission that involves 5 European countries. The project’s objective is to comprehensively outline the entire process of development and ultimately implement mobile technology in practice placements. The project tackles the existing gap by constructing tailored mobile apps designed for students, teachers, tutors, and supervisors within each participating organization. This approach leverages practice-based learning, mobile technology, and technology adoption to enhance the overall educational experience.

**Objective:**

This study aims to introduce mobile technology in clinical practice placements with the goal of facilitating and enhancing practice-based learning. The objective is to improve the overall effectiveness of the process for all stakeholders involved.

**Methods:**

The “4D in the Digitalization of Learning in Practice Placement” (4D Project) will use a mixed methods research design, encompassing 3 distinct study phases: phase 1 (preliminary research), which incorporates focus groups and a scoping review, to define the problem, identify necessities, and analyze contextual factors; phase 2 (collaborative app development), which involves researchers and prospective users working together to cocreate and co-design tailored apps; and phase 3, which involves feasibility testing of these mobile apps within practice settings.

**Results:**

The study’s potential impact will primarily focus on improving communication and interaction processes, fostering connections among stakeholders in practice placements, and enhancing the assessment of training needs. The literature review and focus groups will play a crucial role in identifying barriers, facilitators, and factors supporting the integration of mobile technology in clinical education. The cocreation process of mobile learning apps will reveal the core values and needs of various stakeholders, including students, teachers, and health care professionals. This process also involves adapting and using mobile apps to meet the specific requirements of practice placements. A pilot study aimed at validating the app will test and assess mobile technology in practice placements. The study will determine results related to usability and design, learning outcomes, student engagement, communication among stakeholders, user behavior, potential issues, and compliance with regulations.

**Conclusions:**

Health care education, encompassing disciplines such as medicine, nursing, midwifery, and others, confronts evolving challenges in clinical training. Essential to addressing these challenges is bridging the gap between health care institutions and academic settings. The introduction of a new digital tool holds promise for empowering health students and mentors in effectively navigating the intricacies of the learning process.

**International Registered Report Identifier (IRRID):**

DERR1-10.2196/53284

## Introduction

### Background

Practice-based learning encompasses the educational process in which students acquire knowledge within a service delivery environment tailored to their educational level and competencies. Health profession students, such as nurses, physicians, and physiotherapists, cultivate skills through hands-on experience in various settings. Learning occurs through observation and subsequent participation in clinical tasks.

Conceptually, learners in practice settings acquire “knowledge-in-action” [1] through interaction with experienced professionals, patients/clients, and their peers. For health profession students, practice-based learning provides an opportunity for direct engagement with “real” patients in authentic settings where actual health care is delivered. Health profession students cannot grasp the full complexity of the health care environment until they acquire the specific knowledge, skills, and attitudes that demonstrate competency in their area of practice [2].

The learning process in clinical placements typically involves a diverse array of individuals, including tutors, supervisors, mentors, teachers, and students. Throughout this intricate process, tasks and mentorships must be implemented in a manner that aligns with both educational and clinical processes and is well-embedded in the respective contexts [3].

The learning process, connecting the academic environment with health care centers, encompasses a broad variety of scenarios and contexts. Numerous factors are involved in this complex and challenging practical training for health profession students, affecting both the respective actors and institutions. These factors can result in an inefficient process, causing dissatisfaction and frustration. Much of the effort is directed toward coordinating the involved parties rather than focusing on the learning process itself and its quality.

The interplay between learning and education at the university, along with practical training in placements, constitutes core elements of health care degree programs at universities, such as nursing or medicine [4]. The purpose of education in practice placements is to equip future professionals with the ability to manage their learning, make decisions that enable effective action in their future professional practice, and enhance the quality of care provision [5]. To attain these objectives, practice-based learning becomes indispensable as it necessitates students to scrutinize and assess their patient care, evaluate and integrate scientific evidence, and consistently enhance patient care through ongoing self-evaluation. Students are expected to cultivate skills and habits that enable the practical application of clinical and transversal competencies acquired at the university.

Practice-based learning in health care settings is defined and characterized by several key aspects [6]. These include the contexts in which practices occur [7], their purpose or objective [8], the methods used to assess students [9], the overall commitment within health care degree programs, the practice education model [10-13], and the various actors involved [3,14].

### Elements of Practice Placement in Health Care Education

According to Jokelainen et al [15], the 2 fundamental aspects of mentorship in practice placement involve establishing a supportive learning environment and addressing aspects related to the mentorship process itself. Delving into the establishment of learning support environments, 2 elements are identified as facilitators of learning and indicators of clinical practice environments. These elements are (1) the preparation of the practice setting for learning, which involves planning the training and practice placement, ensuring the implementation of training in the practice setting, and providing opportunities for individualized support during placement; and (2) the organization of interpersonal learning practices, encompassing becoming familiar with the workplace as a work environment, promoting equal participation in practices through teamwork, and collaborating with other stakeholders involved in training.

In delving into the mentorship process within a practice placement, 2 elements that serve as facilitators of learning and indicators of clinical practice placement are recognized. These are (1) facilitating student learning by establishing a supportive learning environment and fostering individualized learning processes; and (2) enhancing the professionalism of students by encouraging the development of professional attributes and identity, ultimately improving the attainment of professional competence.

Furthermore, Thomson et al [16], in a qualitative phenomenological study, identified 5 significant aspects of the experience in tutoring and follow-up in nursing student practice environments. These mentoring and follow-up aspects were (1) being more independent, (2) receiving support, (3) a sense of belonging to the profession, (4) feedback on the learning process, and (5) anticipatory anxiety.

In this intricate scenario involving various actors, institutions, placement contexts, and mentoring approaches, among others, numerous processes are undertaken, requiring substantial resources and time investments. Given that the primary goal of practice-based learning is student learning and the enhancement of their clinical practice, it becomes crucial to develop innovative approaches that can enable more efficient resource management. In this context, the integration of mobile technology in practice settings has the potential to support and enhance students’ learning process while concurrently reducing the resources required for administrative processes in practice placements.

### Introducing Mobile Technology in the Learning Process

Contemporary health systems are shifting toward more integrated and person-centered care models [17], with the use of technology becoming increasingly common in various processes related to care provision. Within this context, health care higher education institutions are incorporating technology into their degree programs with the goal of equipping students with essential skills in digital health [18] and preparing them for their future workplaces. The integration of mobile technologies, coupled with advancements in digital literacy, is expected to empower professionals to confront the intricate challenges presented by contemporary health systems [19]. Additionally, these technologies should facilitate student learning, particularly during clinical practice periods. The education of health students is grounded in the preparation of future professionals capable of navigating this evolving context. Therefore, a key objective of training and learning is the transition from acquiring established knowledge to educating for an unknown future. This shift in learning necessitates the adoption of new approaches and the utilization of innovative teaching and learning technologies [20]. Hence, the utilization of information and communication technologies and Web 2.0 environments in the context of learning during practice placements plays a pivotal role in preparing for this uncertain future in education. Technology-enhanced learning encompasses the application of technology to support any learning-related activity, concentrating on various pedagogical domains that leverage technology [21-23].

The evolution of the Web 2.0 concept has seen a shift in learning from eLearning to mobile learning (mLearning). mLearning is characterized as learning that occurs in diverse contexts, involving social interactions and content consumption, using personal electronic devices as a means of distance education. In this approach, mLearners (students) use educational technology through mobile devices [24] at their convenience [25]. The application and utilization of mLearning in education are contingent on the specific learning needs, context, and objectives to be accomplished [26,27].

In practice placements, where the learning needs involve reinforcing and applying competencies acquired at the university, mLearning serves as a valuable tool for consulting reference materials. Its accessibility virtually anywhere and anytime allows students to enhance their understanding. Furthermore, students can share experiences and knowledge gained in the practice setting with mentors and peers, enabling instant feedback and suggestions. This highly interactive process has demonstrated a 22% reduction in abandonment rates in technical environments, accompanied by an increase in evaluation scores from the 50th percentile to the 70th percentile [28]. In the context of mobility, mLearning facilitates student movement, offering excellent content portability by replacing traditional books and notes with small, personalized devices filled with learning materials. Its convenience stems from its accessibility from almost anywhere.

To effectively tackle current issues in teaching and learning, integrate technologies into their respective practices, and enhance user acceptance, mLearning solutions must be collaboratively designed with all stakeholders. This includes researchers, teachers, students, and administrative staff. Achieving a sustainable digitization and transformation of higher education demands a human-centered approach [29,30] that fosters adoption and ensures a lasting impact on practices. Applying this approach to the digitization and transformation of practice-based learning in health care can aid in comprehending the determinants and factors contributing to the successful introduction of mLearning in practice placements. To bridge the gap between the various actors in these learning contexts (university and clinical practice placement) and enhance the overall experience in practice-based learning in health care settings, it becomes essential to implement mLearning approaches in practice placements and gain insights into their tangible benefits and optimal usage strategies.

This paper outlines the protocol for a study focused on the implementation of mobile technology in practice placements. The study protocol aligns with the research vision of an innovation project in higher education known as the “4D in the Digitalization of Learning in Practice Placement” (4D Project; Multimedia Appendices 1 and 2), involving participants from 5 European countries, namely, Spain, Germany, The Netherlands, Austria, and Poland [31] (Multimedia Appendix 3).

The project aims to fill this gap by creating personalized mobile apps for students, teachers, tutors, and supervisors in each participating organization. This will be achieved through the integration of practice-based reflective learning, mobile technology, and the adoption of technology. The aims are as follows:

- To determine the key factors (barriers, facilitators, and solutions) to introduce mobile technology in practice placements.
- To cocreate design learning practices, materials, and adapt or adopt mLearning technology in practice placements using various co-design methods. This approach aims to respect users’ core values and address their needs.
- To test and assess the introduction of this mobile technology in practice placements in 3 different health institutions in European countries.

## Methods

### Study Design and Methodological Framework

The research will follow a mixed methods research design, incorporating 3 distinct study phases, each utilizing different methodologies.

In phase 1, the focus is on comprehending the facilitators and barriers associated with the integration of mobile technology into clinical education during practice placements for medical and health care students. The goal is to identify potential strategies and approaches to overcome the identified barriers.

During phase 2, the emphasis is on collaborative creation and design with users, involving various design methods. The objective is to cocreate and co-design an mLearning technology specifically tailored for practice placements. This phase aims to incorporate user input and preferences into the development process.

In phase 3, the research moves to the practical testing of the mLearning technology in a real-world environment. The focus is on evaluating the impact, usability, design effectiveness, interactive learning features, and overall user satisfaction. This phase involves assessing how well the developed technology performs in a real setting and gathering feedback to refine and improve its functionality.

### Theoretical Framework

**Figure 1:**
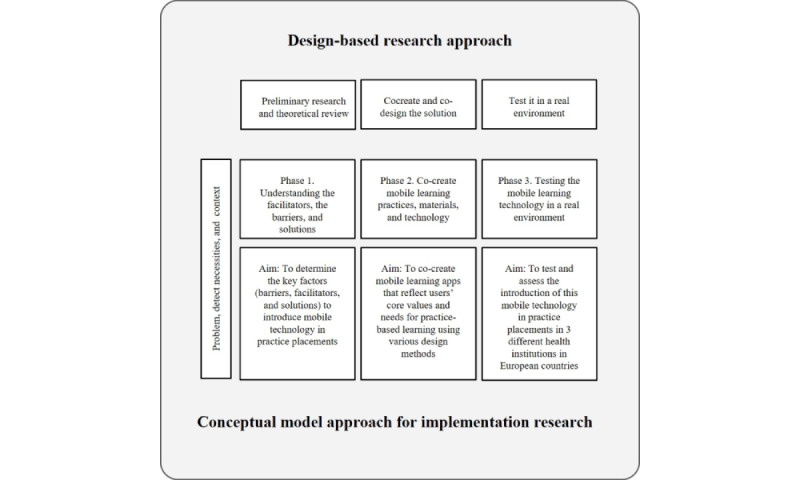
4D Project conceptual model approach for implementation research [35].

The study will be guided by a design-based research framework, which aligns with the principles of design-based research [32-34]. This framework acknowledges that initial research and theoretical review are essential for understanding the problem, identifying needs, and analyzing the context (as seen in phase 1). Subsequently, during the next phase, researchers actively engage in the cocreation of the solution using various design methods (as in phase 2). The final phase involves testing the solution in a real-world environment to assess its impact on students’ learning (as in phase 3). This iterative process allows for the continuous refinement and improvement of the developed solution. The study includes a reflecting phase that directs researchers to reflect on the outcomes and use this reflection to redesign the solution. The iterative research process involves designing, testing, evaluating, and reflecting, leading to a new research cycle aimed at refining and redesigning the solution [35] (Figure 1). This cyclical approach allows for continuous improvement and adaptation based on the insights gained from each iteration.

The following sections detail methodological considerations for each phase:

### Phase 1: Understanding the Facilitators, the Barriers, and Solutions

#### Overview

During this initial phase, the primary objective is to comprehend the facilitators and barriers associated with the integration of mobile technology into clinical education during practice placements for medical and health care students. The focus is on identifying potential solutions to overcome the identified barriers, laying the foundation for subsequent phases of the research.

#### Aim

The aim of this phase is to determine the key factors (barriers, facilitators, and solutions) to introduce mobile technology in practice placements. We describe 3 research questions to ensure and support the objective’s consecution:

- What are the facilitators for introducing mobile technology into clinical education in the practice placement of medical and health care students?
- What are the barriers to introducing mobile technology into clinical education in the practice placement of medical and health care students?
- What are the solutions to overcome barriers for introducing mobile technology into clinical education in the practice placement of medical and health care students?

#### Research Design

To carry out the assessment, we will use 2 methods. First, a scoping literature review will analyze and synthesize existing research evidence. Simultaneously, focus groups will be conducted to capture the perspectives of undergraduate health profession students and stakeholders on the barriers and facilitators associated with the introduction of mobile devices in practice placements. The scoping literature review adheres to the recommendations of the PRISMA-ScR (Preferred Reporting Items for Systematic Reviews and Meta-Analyses Extension for Scoping Reviews) statement [36]. The review process follows the framework established by Arksey and O’Malley [37], further refined by Levac and colleagues [38], encompassing the following stages: (1) identification of research questions; (2) identification of relevant studies; (3) selection of studies; (4) data charting; and (5) collation, summarization, and reporting of results.

Focus groups will be conducted to analyze the barriers, facilitators, and needs associated with introducing mobile technology in practical training for future health care students. This qualitative data collection method aims to offer an in-depth understanding of the phenomena. The findings will be reported following the guidelines outlined in the COREQ (Consolidated Criteria for Reporting Qualitative Research) checklist [39]. The content for the focus groups will be collaboratively developed through consensus among the research team and subject matter experts. Research questions have been formulated in alignment with the research’s purpose by experts representing all partners involved in the 4D Project. This collaborative approach ensures a comprehensive and well-informed set of questions for the focus groups.

#### Sampling Procedures of Focus Groups

Participants for the focus groups will include undergraduate health care students and stakeholders from Tecnocampus, Pompeu Fabra University, the Medical University of Lublin (Poland), the University of Duisburg Essen (Germany), and the Germans Trias i Pujol University Hospital (Spain). The selection of participants will be carried out using purposive sampling with the aim of achieving maximum variation. Students eligible for participation should be enrolled in degree programs such as nursing, medicine, physiotherapy, or midwifery. Stakeholders will also be included based on the following criteria: involvement in the practical training of future nurses, midwives, physiotherapists, or doctors in roles such as clinical mentors; link teachers; practical training coordinators; hospital ward managers; or staff in the nursing, midwifery, and medical fields.

In each country, we have scheduled 2 focus groups with 8-10 participants each. Overall, we plan to conduct 3 focus groups with undergraduate students (nurses, physiotherapists, midwives, or medical students) and 3 focus groups with stakeholders, including clinical mentors, link teachers, practical training coordinators, hospital ward managers, staff, and other relevant stakeholders.

#### Analysis

In the scoping literature review, the study screening will follow a structured process [40] involving a sequential review by title, abstract, and full text. Two pairs (pair 1: BD and Agnieszka Chrzan-Rodak; pair 2: Cristina Casanovas Cuéllar and Ariadna Huertas Zurriaga) of experienced researchers will participate in this screening process. Initially, study titles will undergo independent screening by paired reviewers to identify studies that meet the inclusion criteria. Subsequently, researchers will assess the abstracts for inclusion, reviewing a distinct group of articles from the previous step. Following this, the researchers will rigorously evaluate the full text of the remaining articles, strictly adhering to the inclusion and exclusion criteria. In each round, a third researcher will scrutinize the work of each reviewing pair for potential errors. In case of any disagreement between the paired researchers, the document will be referred to the next researcher in the process to mitigate deselection bias. The third researcher will additionally assess the level of agreement between review pairs for both the title and abstract phases, aiming for a 95% agreement level.

The analysis of focus group data will adhere to Koole’s FRAME (Framework for the Rational Analysis of Mobile Education) model [41]. The FRAME model delineates 3 principal aspects influencing mLearning and specifies factors (device usability, interaction learning, and social technology) that impact the successful integration of these aspects. For this study, the FRAME model will be adapted to encompass all the findings generated throughout the research.

The initial phase of analysis involves 1 of the researchers (BD) reading the transcripts multiple times to gain familiarity with the content and identify initial units of meaning. Subsequently, the texts will be coded using descriptive codes based on their content. These codes will then be organized into categories according to similarities in the codes. At this juncture, the preliminary results will be discussed within the research group. Through reflective thinking and critical reasoning, adjustments will be made until a consensus is reached. The themes derived from the analysis will be organized under the learning aspects outlined in Koole’s original model. Subsequently, the resulting report will be discussed with 2 participants to validate the meaning and coherence of the interpretations. To enhance the trustworthiness of the data, considerations will be given to credibility, dependability, conformability, and transferability [42]. The participants for this discussion will be selected through purposive sampling with maximum variation to bolster credibility. The information from the focus groups will undergo thorough examination by at least two members (SH and CMG) of the research team, and the results will be compared to evaluate dependability. To ensure conformability, all information provided by participants will be presented transparently. For the transferability of the study’s data, a comprehensive and detailed description of the data and context will be provided.

### Phase 2: Cocreate Mobile Learning Practices, Materials, and Technology

#### Overview

This phase is dedicated to collaboratively creating mLearning practices, materials, and technology for practice-based learning using various (co-)design methods.

#### Aim

The objective is to cocreate mLearning apps that align with users’ core values and needs for practice-based learning. This involves collaborative design and production of mLearning practices and materials. Additionally, it includes the adaptation and appropriation of existing mobile apps to bridge the gap between the academic and practice contexts in health care practice placements.

#### Research Design

This section of the study utilizes collaborative design methods to comprehend the core values and needs of health care students and stakeholders. The goal is to develop embedded mLearning apps for practice placements. To facilitate the coordination of the innovation process within the design team and with domain representatives, the university innovation canvas (UIC) is used. The UIC elucidates the key factors fostering digital transformation and sustainable innovation. This will help to plan the selection and application of more specific co-design methods and tools such as the value proposition canvas (VPC), personas, storytelling, and use case definitions.

#### University Innovation Canvas and Value Proposition Canvas

The UIC draws inspiration from the business model canvas by Osterwalder and Pigneur [43] and the lean canvas by Maurya [44]. While the business model canvas primarily explores the creation of value for businesses, the UIC [30] is designed to reflect how “value” is generated within a university setting. This adaptation aligns the canvas with the specific context and objectives of fostering innovation within the university environment. In our scenario, the aim is to establish a shared understanding of enhancing collaboration between universities and their affiliated practice placement organizations, involving all relevant stakeholders (eg, students, teachers, and nurses). The UIC comprises 11 elements organized into 3 dimensions: the technology-enhanced learning concept (value creation), stakeholder relationships (value delivery), and foundation and scaling (value capture). This framework provides a structured approach to addressing key aspects of innovation and value creation in the university setting. The specific elements and dimensions within the canvas serve as tools for various stakeholders from diverse contexts to refine their collective focus. They facilitate reflection on crucial factors related to planned sustainable innovation, particularly in the context of enhancing practice placements. The canvas provides a structured framework that encourages stakeholders to align their perspectives and contribute to the development of innovative solutions.

To precisely define the value proposition within the UIC and align it with the technologies available for adoption within the consortium, we opted for the VPC [45]. This tool concentrates on how to generate value for all stakeholders involved (eg, students, teachers, nurses), or more precisely, how these stakeholders can benefit from the anticipated learning intervention. The VPC offers a structured approach to articulate and understand the unique value that the proposed innovations bring to each stakeholder. The VPC consists of 2 parts: First, the *stakeholder profile*, which aims at (1) describing the things or tasks they must do during their work; (2) related pains during the work including risks, potential bad outcomes, or obstacles related to their job; and (3) desired gains including the outcomes and benefits they would like to have. Second, the *value map*, which aims at creating value for the stakeholders by identifying the following: (1) pain relievers in the form of an innovation to reduce/eliminate the stakeholder’s pains, (2) innovations that could be built around the value proposition, and (3) gain creators that describe how the innovation creates gains for the stakeholders.

#### Participants

The participants involved in this process are all members of the 4D Project partner teams, particularly those working at universities or in hospitals. This inclusive approach aims to gather perspectives and insights from both the education side at the university and the practice placements, ensuring a comprehensive representation of stakeholders involved in the project.

#### Procedure and Analysis

The application of the UIC and VPC will follow a 2-step approach, commencing with a “top-down” approach and subsequently transitioning to a “bottom-up” approach. In the initial step (“top-down” approach), the UIC will be distributed to all 4 distinct practice placements. The stakeholders engaged in the practice placements will receive an introduction to the UIC along with a clear explanation of the purpose behind this activity. The objective is to illuminate the addressed problems, identify value propositions and measures, and identify all involved stakeholders from various perspectives. After the completion of the UICs for each of the 4 practice placements, the analysis will focus on uncovering commonalities across all of them. In the subsequent step (ie, the “bottom-up” approach), 3 of the identified commonalities will undergo a more detailed analysis. Hence, in a face-to-face setting, the pertinent stakeholders will be invited to vote for the most significant commonalities and collaboratively complete the VPC. The objective is to explore, from a value proposition perspective, how potential innovations could assist them in creating value for their stakeholders (eg, students, lecturers, and nurses). After the completion of the VPCs, the input will be analyzed and used to generate new UICs. This process aims to establish a shared understanding of possible solutions that align with all 4 practice placements and to emphasize how such innovations can be applied across all 4 settings. Based on the outcomes, subsequent co-design activities using personas, storytelling, and use case definition will be used to further refine the elicited innovation. This may involve creating mock-ups and developing application scenarios in practice. The process includes collaborative efforts to cocreate mLearning practices, materials, and suitable tools within the consortium and with all stakeholders. The overarching goal is to support the practice-based learning of health care professionals in education through innovative and tailored solutions.

### Phase 3: Testing the Mobile Learning Technology in a Real Environment

#### Overview

This phase is dedicated to testing the mLearning technology in a real environment, evaluating its impact, usability, design, interactive learning, social technology, and overall satisfaction.

#### Aim

The objective is to test and assess the introduction of this mobile technology in practice placements within 3 distinct health institutions located in Spain, Germany, and Poland.

#### Research Design

In this phase of the study, the descriptive method, specifically a survey, will be used. The underlying premise is that the primary users, namely, the students and clinical mentors, can provide valuable information on aspects related to the use of mobile devices in a practice placement.

The proposed data collection method involves designing and crafting a questionnaire to ensure a comprehensive exploration of the subject. A self-administered questionnaire will be formulated, comprising distinct sections, including a participant information sheet; consent form; demographics information; and segments focusing on usability, design interaction, learning, social technology, and satisfaction. The questionnaire has been created by the authors of the study and draws upon the insights gained from the literature review [41,46,47] and the outcomes obtained in phase 2. Response options will consist of a 5-point Likert scale (ranging from 5=strongly agree to 1=strongly disagree), short responses, or free-text answer options. To assess the feasibility of the questionnaire, it will undergo a pilot phase with a group of students and stakeholders engaged in clinical education. Subsequently, the questionnaire will be revised, if necessary, based on the feedback received during the pilot testing. All participants will be provided with information about the study, including the study protocol, a digital consent form, and a questionnaire. Only those who agree to participate in the study will be directed to the questionnaire. Participants retain the right to withdraw from the study at any point. The online survey is estimated to take approximately 15 minutes to complete. The collected data will be securely stored on a password-protected computer, with access restricted to designated research staff or other authorized personnel who are obligated to maintain the confidentiality of the information. It is important to note that the data will be anonymized.

#### Participants

The study sample will consist of participants from Tecnocampus, Pompeu Fabra University (Spain), the Medical University of Lublin (Poland), the University of Duisburg Essen (Germany), and the Germans Trias i Pujol University Hospital (Spain). The questionnaire will be completed by students, teachers, tutors, and supervisors who use the developed apps. Moreover, all other individuals engaged in clinical education (practice placement) will be invited to participate in the study.

#### Analysis

The survey will be designed and implemented using REDCap (Research Electronic Data Capture), a secure, web-based application specifically designed to facilitate data capture for research studies [48].

The analysis in this phase will use inferential statistics to draw conclusions regarding the integration of mobile technology in practical placements. The statistical package used for this analysis will be SPSS, version 23.0 (SPSS Inc.). However, we do not exclude the possibility of using MS Excel (Microsoft Corporation) to facilitate and expedite the data collection and analysis process.

The methodology for describing and analyzing open free-text answers (unstructured data) will involve the use of Dcipher Studio (Dcipher Analytics), an artificial intelligence–powered tool designed to identify topics and sentiments in free-form text responses [49]. Using sentiment analysis and tonality detection will prove valuable in quantifying the emotional tones expressed in the responses.

The analysis of mobile app usage will involve reviewing computer-generated event logs to identify bugs and security threats, ensuring compliance with regulations, and gaining insights into user behavior.

### Ethical Issues

At every stage of the planned research, strict adherence to the ethical principles outlined in the Declaration of Helsinki will be maintained. In research involving individuals, such as students and stakeholders, each partner will submit an application containing a comprehensive description of the research protocol to the relevant Ethics Committee. The focus groups and questionnaire studies will adhere to the principles of voluntary participation, anonymity, and respect for the decision to withdraw from the study at any stage. The gathered data will be used solely for purposes associated with the implementation of the 4D Project.

### Ethics Approval

The study has received approval from the ethics committees of the respective institutes in each country: the Bioethical Commission at the Medical University of Lublin in Poland (protocol code KE-0254/152/06/2022 approved on June 30, 2022), the University of Duisburg Essen’s Ethics Committee in Germany (protocol code 22-10783-BO approved on October 19, 2022), and the Fundació Tecnocampus Mataró-Maresme's Ethics Committee in Spain (protocol code CEI2/2022 approved on October 7, 2022). Informed consent will be obtained from all study participants.

## Results

### Phase 1: Understanding Facilitators and Barriers

The scoping literature review commenced in April 2022 and concluded in July 2022, while the focus groups were conducted from October to December 2022. It is anticipated that the outcomes of phase 1 will reveal (1) the identification of factors supporting the successful integration of mobile technology in clinical education for medical and health care students; (2) the acknowledgment of obstacles and challenges impeding the effective use of mobile technology in practice placements; (3) practical solutions proposed to address the identified barriers and challenges, and (4) insights derived from the focus groups, which will provide a deep understanding of the perspectives and experiences of health care students and stakeholders regarding the integration of mobile technology.

### Phase 2: Cocreating Mobile Learning Practices

The design process commenced in July 2022 and concluded in November 2023. The outcomes of phase 2 revealed (1) the development of innovative and customized mLearning apps that resonate with the core values and requirements of health care students and stakeholders; (2) the adaptation and proficient utilization of preexisting mobile apps to fulfill the distinct needs of practice placements; and (3) the recognition of how collaboratively crafted mLearning practices can yield value for a diverse range of stakeholders, encompassing students, educators, and health care professionals.

### Phase 3: Testing Mobile Learning Technology

The pilot study is set to commence in January 2024. It is anticipated that the outcomes of phase 3 will reveal (1) results pertaining to the usability and design of the collaboratively created mLearning apps obtained through surveys; (2) the impact of mobile technology integration on various aspects including learning outcomes, student engagement, and communication among stakeholders; (3) an examination of the emotional tones expressed by participants in relation to the implementation of mobile technology; and (4) findings from the analysis of computer-generated event logs from mobile apps, providing insights into user behavior, identifying potential issues, and ensuring compliance with regulations.

## Discussion

### Principal Findings

The primary objective of this study is to bridge the gap in practice-based learning by developing personalized mobile apps tailored for diverse actors and stakeholders. The study aims to assess the integration of mobile technology in practice placements within European health care institutions.

This study aims to showcase the transformative potential of mobile technology in reshaping practice placements within health care education. Through targeted efforts to overcome significant barriers, engaging stakeholders in co-design processes, and integrating mobile apps, the study will concentrate on enhancing both the learning and administrative aspects of practice placement. The overarching goal is to ensure the effective clinical education of health care students in higher education.

Identifying key factors, including barriers, facilitators, and potential solutions, for the introduction of mobile technology in practice placements will unveil a spectrum of elements. Literature reviews and insights from focus groups suggest that factors facilitating the use of mLearning in practice placements may include improved access to clinical resources; enhanced communication and collaboration among health care professionals, students, and stakeholders; and the facilitation of self-directed learning. Barriers to the integration of mobile technology in practice placements are concerns about the design of the product being beyond the control of learners and their teaching staff. Additionally, challenges arise when cultural acceptance and adherence to social norms regarding the use of mobile devices in clinical settings are not taken into account. Moreover, the absence of clear policies further contributes to impediments in this context [50-52].

The co-design of an mLearning app, aligning with users’ core values and needs, is crucial for the effective adoption of mobile technology in practice-based learning [53]. The outcomes will offer valuable insights into users’ needs, values, and preferences, guiding the design process to ensure a user-centered application [54].

Ultimately, the testing and assessment of mobile technology in clinical practice placements are poised to yield promising results across various dimensions, including usability and design, learning outcomes, student engagement, communication among stakeholders, user behavior, identification of potential issues, and compliance with regulations. The integration of mobile apps holds the potential to have a positive impact on the learning process, streamlining day-to-day clinical practice, and enhancing value-based activities.

The impact of this study underscores the necessity for sustained investment in technology-enhanced learning and the crucial role of user-centered design in health care education. Extending beyond the immediate scope of this study, the findings will emphasize broader implications for health care education, particularly in clinical practice placements.

### Limitations

It is important to note that the findings of this study may have limited generalizability beyond the participating European countries due to variations in educational contexts and health care systems. Moreover, the assessment of learning outcomes and intervention effectiveness in complex educational settings poses challenges, potentially impacting the comprehensiveness and quantifiability of our results.

### Conclusions

The 4D Project endeavors to address the existing gap between academic contexts and clinical placements in higher education. Its objective is to introduce mobile technology as a solution to enhance the learning process in this context. The project actively supports the examination of communication and interaction processes among various stakeholders within and across different organizations and countries. This is achieved through a meticulous methodology used in the design of a mobile app.

The study will use a multiphase approach. Initially, a systematic review and qualitative methodology involving focus groups will be used to identify barriers and facilitators to the introduction of mobile technology in practice placements. Subsequently, a design-based research methodology will be implemented, enabling the cocreation of mLearning apps that align with users’ core values and needs for practice-based learning. Finally, a pilot study using both quantitative and qualitative methods will be conducted to validate and test the mLearning technology in a real-world environment. This phase will evaluate its impact, usability, design, interactive learning, social technology aspects, and user satisfaction.

The long-term benefits of the “4D Project” are centered around improving communication, interaction, and collaboration among the diverse stakeholders engaged in the educational process. By addressing current challenges in practice placements, the project aims to fortify the connection between these stakeholders in the digital era. It seeks to foster new forms of collaboration through the strategic integration of mobile technology.

Health care education, encompassing disciplines such as medicine, nursing, midwifery, and others, encounters ongoing challenges in clinical training. The imperative task of bridging the divide between health care institutions and academic settings is crucial. The introduction of a novel digital tool holds the potential to empower health students and mentors, providing a means to navigate the complexities inherent in the learning process.

## Appendix

Multimedia Appendix 1 Selected projects 2021-KA220-HED-s.

Multimedia Appendix 2 Eligibility outcome for the 4D Project. 4D Project: 4D in the Digitalization of Learning in Practice Placement.

Multimedia Appendix 3 4D Project grant agreement. 4D Project: 4D in the Digitalization of Learning in Practice Placement.

## Abbreviations

4D Project: 4D in the Digitalization of Learning in Practice Placement
COREQ: Consolidated Criteria for Reporting Qualitative Research
FRAME: Koole’s Framework for the Rational Analysis of Mobile Education
mLearning: mobile learning
PRISMA-ScR: Preferred Reporting Items for Systematic Reviews and Meta-Analyses Extension for Scoping Reviews
REDCap: Research Electronic Data Capture
UIC: university innovation canvas
VPC: value proposition canvas
