# Supplementary material for: Connecting Actors With the Introduction of Mobile Technology in Health Care Practice Placements (4D Project): Protocol for a Mixed Methods Study
Source: JMIR Res Protoc. 2024 Feb 8;13:e53284. doi: 10.2196/53284 (PMC10884912; doi:10.2196/53284)
Supplement: Multimedia Appendix 1 [file resprot_v13i1e53284_app1.pdf]

Programa Erasmus+

**Convocatoria**	2021
**Acción clave**	KA2
**Ronda**	1
**Anexo III.1**	Asociaciones de cooperación en Educación Superior (KA220-HED). Solicitudes seleccionadas.
**Total**	9.920.331,00 €

Nº	Nº PROYECTO	Nº SOLICITUD	TITULO DEL PROYECTO	OID	NOMBRE DE LA ORGANIZACIÓN	LOCALIDAD	COMUNIDAD AUTÓNOMA	PRESUPUESTO ASIGNADO
1	2021-1-ES01-KA220-HED-000029950	KA220-HED-62787AB2	Universidades por el Desarrollo Sostenible (UDS)	E10208678	UNIVERSIDAD PABLO DE OLAVIDE	SEVILLA	Andalucía	371.487,00 €
2	2021-1-ES01-KA220-HED-000034146	KA220-HED-F5265FF4	360 REWIN-REsilient immigrant Women interventions for INclusion	E10206524	UNIVERSIDAD DE BURGOS	BURGOS	Castilla y León	394.370,00 €
3	2021-1-ES01-KA220-HED-000031128	KA220-HED-57E82F65	ENHANCE – Enhancing Career and Service Learning in Higher Education	E10208817	UNIVERSIDAD DE VALLADOLID	VALLADOLID	Castilla y León	242.508,00 €
4	2021-1-ES01-KA220-HED-000027570	KA220-HED-4569865C	A new educational model for acquisition of sustainability competences through service-learning	E10209047	UNIVERSIDAD PUBLICA DE NAVARRA	PAMPLONA	Navarra	285.882,00 €
5	2021-1-ES01-KA220-HED-000032189	KA220-HED-13653E18	Digital Electronics Collaborative Enhanced Learning	E10208584	UNIVERSIDAD DE ALCALA	ALCALA DE HENARES/MADRID	Comunidad de Madrid	277.505,00 €
6	2021-1-ES01-KA220-HED-000022911	KA220-HED-C95C4048	A Connective, Inclusive and Smart digital Competence-building Framework	E10209030	UNIVERSIDAD REY JUAN CARLOS	MOSTOLES	Comunidad de Madrid	200.315,00 €
7	2021-1-ES01-KA220-HED-000035794	KA220-HED-7A2C0B59	Technology for Healthcare education using smart gamification	E10208943	UNIVERSIDAD DE LA IGLESIA DE DEUSTO ENTIDAD RELIGIOSA	BILBAO	País Vasco	214.623,00 €
8	2021-1-ES01-KA220-HED-000031173	KA220-HED-2364ED4D	Propuesta de Intervención para el desarrollo de la Resiliencia en la Educación Superior. Superación de adversidades.	E10208989	UNIVERSIDAD DE GRANADA	GRANADA	Andalucía	291.138,00 €
9	2021-1-ES01-KA220-HED-000031153	KA220-HED-2380A790	GIRLS TEAM UP!	E10069994	FUNDACIÓN DIOCESANA DE ENSEÑANZA SANTOS MÁRTIRES DE CÓRDOBA	CÓRDOBA	Andalucía	220.430,00 €
10	2021-1-ES01-KA220-HED-000032185	KA220-HED-3F8A996D	Promoting digital entrepreneurial mindsets in Higher Education	E10209448	UNIVERSITAT AUTONOMA DE BARCELONA	CERDANYOLA BARCELONA	Cataluña	308.990,00 €
11	2021-1-ES01-KA220-HED-000030125	KA220-HED-C047AAD1	Habilidades transversales en la Inteligencia Artificial Aplicada	E10209121	UNIVERSIDAD DE MALAGA	MALAGA	Andalucía	372.615,00 €
12	2021-1-ES01-KA220-HED-000032088	KA220-HED-0E728979	EUROPEAN HEALTH CARE FINAL DISSERTATION: a digital, international, and collaborative co-designed model to address health care societal challenges	E10208601	FUNDACIO UNIVERSITARIA BALMES	VIC BARCELONA	Cataluña	368.429,00 €
13	2021-1-ES01-KA220-HED-000027552	KA220-HED-665E0602	Inclusive Comprehensive Internationalisation: Enhancing global learning opportunities for ALL students	E10208977	UNIVERSITAT ROVIRA I VIRGILI	TARRAGONA	Cataluña	327.056,00 €
14	2021-1-ES01-KA220-HED-000030297	KA220-HED-C7530EB2	DIGital competences for engaGING future educators	E10209121	UNIVERSIDAD DE MALAGA	MALAGA	Andalucía	213.294,00 €

Código seguro de Verificación : GEN-da9c-c1b0-e8d9-4f5f-a6a0-5ed6-893a-c707 | Puede verificar la integridad de este documento en la siguiente dirección : https://sede.administracion.gob.es/pagSedeFro...

CSV : GEN-da9c-c1b0-e8d9-4f5f-a6a0-5ed6-893a-c707
DIRECCIÓN DE VALIDACIÓN : https://sede.administracion.gob.es/pagSedeFront/servicios/consultaCSV.htm
FIRMANTE(1) : ALFONSO GENTIL | FECHA : 29/12/2021 14:24 | Sin acción específica

Programa Erasmus+

Convocatoria 2021
Acción clave KA2
Ronda 1
Anexo III.1 Asociaciones de cooperación en Educación Superior (KA220-HED). Solicitudes seleccionadas.
Total 9.920.331,00 €

Nº	Nº PROYECTO	Nº SOLICITUD	TITULO DEL PROYECTO	OID	NOMBRE DE LA ORGANIZACIÓN	LOCALIDAD	COMUNIDAD AUTÓNOMA	PRESUPUESTO ASIGNADO
15	2021-1-ES01-KA220-HED-000032107	KA220-HED-D4A904A5	STEAM-Active	E10208839	UNIVERSIDAD DEL PAIS VASCO/ EUSKAL HERRIKO UNIBERTSITATEA	LEIOA	País Vasco	301.733,00 €
16	2021-1-ES01-KA220-HED-000023527	KA220-HED-4845EB35	University and Business Inclusive Digital Learning Coaches	E10209100	UNIVERSIDAD DE ALICANTE	ALICANTE	Comunidad Valenciana	161.239,00 €
17	2021-1-ES01-KA220-HED-000031988	KA220-HED-B2F4704B	University cooperation for promoting the GREEN transition and sustainable practices in education and training	E10209013	UNIVERSIDAD DE ALMERIA	ALMERIA	Andalucía	346.637,00 €
18	2021-1-ES01-KA220-HED-000027496	KA220-HED-0538081F	4D in the Digitalisation of Learning in Practice Placement	E10158035	FUNDACIO TECNOCAMPUS MATARO-MARESME	MATARO	Cataluña	377.840,00 €
19	2021-1-ES01-KA220-HED-000032243	KA220-HED-8BDD6060	PLURIMATHS: Learning Mathematics Through Pluriliteracies	E10208905	UNIVERSIDAD DE CORDOBA	CORDOBA	Andalucía	222.988,00 €
20	2021-1-ES01-KA220-HED-000032096	KA220-HED-E327505D	NUEVO MODELO DE CUALIFICACIÓN HÍBRIDA Y MULTINIVEL (FORMACIÓN PROFESIONAL + UNIVERSIDAD + EMPRESA) PARA NUEVAS NECESIDADES DE EMPLEO Y PERFILES EMERGENTES	E10211800	FUNDACION SAN VALERO	ZARAGOZA	Aragón	245.927,00 €
21	2021-1-ES01-KA220-HED-000030418	KA220-HED-3EC00D34	Circular & Socio-Civic Learning Hub	E10209373	UNIVERSIDAD POLITECNICA DE MADRID	MADRID	Comunidad de Madrid	367.035,00 €
22	2021-1-ES01-KA220-HED-000023320	KA220-HED-061587F2	Comunidad de Aprendizaje Universitaria para la Inclusión y la No discriminación	E10208679	UNIVERSIDAD DE SALAMANCA	SALAMANCA	Castilla y León	387.637,00 €
23	2021-1-ES01-KA220-HED-000030446	KA220-HED-726FE527	Innovation Lab: Transform your teaching with the Digital Advisor Toolkit for Higher Education	E10208658	UNIVERSIDAD DE MURCIA	MURCIA	Región de Murcia	227.681,00 €
24	2021-1-ES01-KA220-HED-000023306	KA220-HED-EE28FADA	Educando en Sexualidad: Avance para la Salud Europea	E10208621	UNIVERSIDAD DE CASTILLA - LA MANCHA	CIUDAD REAL	Castilla la Mancha	276.134,00 €
25	2021-1-ES01-KA220-HED-000027551	KA220-HED-BE8FD875	European network in D-flexible teaching	E10208821	UNIVERSIDAD NACIONAL DE EDUCACION A DISTANCIA	MADRID	Comunidad de Madrid	309.565,00 €
26	2021-1-ES01-KA220-HED-000023112	KA220-HED-A307EDB9	INTERNATIONAL WORK BASED LEARNING IN HIGHER EDUCATION	E10208943	UNIVERSIDAD DE LA IGLESIA DE DEUSTO ENTIDAD RELIGIOSA	BILBAO	País Vasco	292.222,00 €
27	2021-1-ES01-KA220-HED-000027588	KA220-HED-78C31409	Leveraging Individual SDG Contributions by University Staff	E10209101	UNIVERSITAT DE GIRONA	GIRONA	Cataluña	348.871,00 €
28	2021-1-ES01-KA220-HED-000032139	KA220-HED-D7CD87AA	JOIN-RISe Joint development of innovative blended learning in STEM curricula based on SDGs for a resilient, inclusive and sustainable education	E10206524	UNIVERSIDAD DE BURGOS	BURGOS	Castilla y León	381.290,00 €

Código seguro de Verificación : GEN-da9c-c1b0-e8d9-4f5f-a6a0-5ed6-893a-c707 | Puede verificar la integridad de este documento en la siguiente dirección : https://sede.administracion.gob.es/pagSedeFro...

CSV : GEN-da9c-c1b0-e8d9-4f5f-a6a0-5ed6-893a-c707
DIRECCIÓN DE VALIDACIÓN : https://sede.administracion.gob.es/pagSedeFront/servicios/consultaCSV.htm
FIRMANTE(1) : ALFONSO GENTIL | FECHA : 29/12/2021 14:24 | Sin acción específica

Programa Erasmus+

Convocatoria 2021
Acción clave KA2
Ronda 1
Anexo III.1 Asociaciones de cooperación en Educación Superior (KA220-HED). Solicitudes seleccionadas.
Total 9.920.331,00 €

Nº	Nº PROYECTO	Nº SOLICITUD	TITULO DEL PROYECTO	OID	NOMBRE DE LA ORGANIZACIÓN	LOCALIDAD	COMUNIDAD AUTÓNOMA	PRESUPUESTO ASIGNADO
29	2021-1-ES01-KA220-HED-000032075	KA220-HED-8DC1CB23	Circular economy in fibrous composites and technical textiles through the use of virtual laboratories - CircuText	E10208835	UNIVERSITAT POLITECNICA DE VALENCIA	VALENCIA	Comunidad Valenciana	263.932,00 €
30	2021-1-ES01-KA220-HED-000032084	KA220-HED-6DD5875E	Inclusive Higher Education Systems for students with intellectual disabilities (IHES)	E10208678	UNIVERSIDAD PABLO DE OLAVIDE	SEVILLA	Andalucía	306.756,00 €
31	2021-1-ES01-KA220-HED-000032193	KA220-HED-E4CC1C20	Short Learning Programme for ARt & DEsign & Sustainability with special focus on environment and climate change	E10128869	DOCUMENTA CREACIONES MULTIMEDIA AVANZADAS SL	BARCELONA	Cataluña	198.340,00 €
32	2021-1-ES01-KA220-HED-000023469	KA220-HED-C1B304CC	Plurilingual Phraseology : Learning multiword units through English	E10208658	UNIVERSIDAD DE MURCIA	MURCIA	Región de Murcia	249.042,00 €
33	2021-1-ES01-KA220-HED-000022963	KA220-HED-BB0397DB	Digitalisation of multilingual programs in the EHEA	E10208939	UNIVERSIDAD DE HUELVA	HUELVA	Andalucía	228.440,00 €
34	2021-1-ES01-KA220-HED-000032074	KA220-HED-1F1438AE	Digital Lab for Education in Dietetics combining Experiential Learning and Community Service	E10050441	Fundación Universidad Europea del Atlántico	Santander	Cantabría	338.380,00 €

Código seguro de Verificación : GEN-da9c-c1b0-e8d9-4f5f-a6a0-5ed6-893a-c707 | Puede verificar la integridad de este documento en la siguiente dirección : https://sede.administracion.gob.es/pagSedeFro...

CSV : GEN-da9c-c1b0-e8d9-4f5f-a6a0-5ed6-893a-c707
DIRECCIÓN DE VALIDACIÓN : https://sede.administracion.gob.es/pagSedeFront/servicios/consultaCSV.htm
FIRMANTE(1) : ALFONSO GENTIL | FECHA : 29/12/2021 14:24 | Sin acción específica
