# Supplementary material for: Connecting Actors With the Introduction of Mobile Technology in Health Care Practice Placements (4D Project): Protocol for a Mixed Methods Study
Source: JMIR Res Protoc. 2024 Feb 8;13:e53284. doi: 10.2196/53284 (PMC10884912; doi:10.2196/53284)
Supplement: Multimedia Appendix 2 [file resprot_v13i1e53284_app2.pdf]

Resultado de la elegibilidad y/o evaluación de la calidad del proyecto

Criterio: Calidad del diseño del proyecto
Puntuación: 12.0
Comentario:

En cuanto a los aspectos positivos, se destaca que el programa de trabajo se expone de forma clara y exhaustiva, identificando las diferentes fases del mismo y los socios implicados. Además, la propuesta es consistente atendiendo a los objetivos que se plantea y las actividades que se incluyen para su consecución. También los resultados definidos en el proyecto son pertinentes y se han descrito de forma adecuada. El proyecto se organiza alrededor de paquetes de trabajo, lo que se valora positivamente. Los paquetes de trabajo y el calendario son eficaces y están cuidadosamente preparados y descritos. Además, existe un plan previo de distribución de tareas entre las organizaciones, así como de arreglos administrativos. En los resultados, la división del trabajo, las tareas que conllevarán la consecución de los mismos y la metodología aplicada están descritas con detalle. También se valora positivamente el uso de la plataforma Erasmus+ Project Results Platform, en cuyo uso los socios tienen experiencia, como herramienta de trabajo y para la búsqueda de stakeholders. Respecto a la temática de las actividades de formación, esta es pertinente dentro de los objetivos del proyecto y adecuada en número y perfil con los participantes. Se realizará un workshop de validación, aplicabilidad y formación de los resultados obtenidos (i.e. la app). Este workshop incluye una variedad de sectores: profesores y formadores, profesionales de la salud, alumnos, compañías y startups. No obstante, se aprecian algunas **debilidades en la solicitud de subvención que podrían restar calidad a la misma. La principal es que el PR3 ?Evaluation and good practices on introducing technology in practice placement. "Pilot study results? no puede ser considerado como un resultado válido del proyecto en su forma actual ya que éste no es susceptible de provocar un impacto positivo real y sustancial en las organizaciones participantes. Se debe reestructurar como una Guía de buenas prácticas ?Guide of good practices on introducing technology in practice placement?.** Otra de las **debilidades es que el plan de control de gasto, así como en el plan de control de los plazos y consecución de los hitos no se detallan suficientemente.** Asimismo, **se aprecia la necesidad de que la propuesta identifique de manera pormenorizada los riesgos potenciales que pueden darse durante la ejecución el proyecto, indicando las medidas previstas para su control. Por último, las reuniones transnacionales, 7 en total, no se consideran coste-efectivas ya que no está justificado que se subvencione a más de un participante por socio. Asimismo, sólo están permitidas 6 reuniones transnacionales, la número 6, relacionada con la diseminación, podría llevarse a cabo de manera virtual con éxito, por lo que se propone su conversión a remota. Entre los aspectos de mejora, se recomienda disminuir el número de reuniones presenciales a 4, y realizar las restantes de manera virtual como medida de sostenibilidad medioambiental.**

Criterio: Calidad de la asociación y de los mecanismos de cooperación
Puntuación: 16.0
Comentario:

En cuanto a los aspectos positivos, destaca que la composición del equipo es adecuada, ya que todos los socios tienen experiencia en alguno de los aspectos que desarrolla el proyecto y sus perfiles son bastante complementarios y compatibles con los objetivos planteados, y con la temática propuesta. También se valora positivamente que la experiencia previa de las organizaciones implicadas es muy alta, tanto en la temática plateada por la propuesta como en otras similares. Además, la propuesta muestra claramente cómo la actividad habitual de las organizaciones participantes está relacionada y se pondrá en práctica dentro del ámbito de la solicitud. Asimismo, el consorcio es multisectorial al estar formado por asociaciones de ámbitos variados y relevantes para la propuesta. Se combinan universidades, dos centros de investigación y uno de ellos asociado a un hospital, y además se incorpora una start-up especialista en software-as-a-service, que proporciona una plataforma de herramientas del aprendizaje. A su vez, las

universidades participantes combinan las áreas de ciencias de la salud y de programación e informática. Alguna tiene experiencia previa en el desarrollo de mobil teaching Apps. Adicionalmente, se valora positivamente la relación con socios no formales relacionadas con ciencias de la salud, así como el contacto con asociaciones de estudiantes, asociaciones de enfermería etc. Además, dos de los socios, INSTITUT DE INVESTIGACIO EN CIENCIES DE LA SALUT GERMANS TRIAS I PUJOL y KUBIFY BV, son nuevos en la Acción lo que se valora positivamente. **Se aprecian algunas debilidades en la solicitud de subvención que podrían restar calidad a la misma, como son las relacionadas con una ligera sobrecarga de trabajo por parte del coordinador del proyecto. Además, los canales de comunicación entre socios no se encuentran muy detallados. Se comenta en la propuesta que se usará un repositorio de documento, como plataforma de comunicación interna, pero no se da ningún detalle de la misma ni otras herramientas adicionales. Como aspecto de mejora sería recomendable proporcionar un mayor detalle de las personas involucradas en el proyecto del socio UNIVERSITAET DUISBURG-ESSEN.**

Criterio: Pertinencia
Puntuación: 30.0
Comentario:

La solicitud cubre claramente tres prioridades, una horizontal y dos sectoriales del Programa Erasmus+ y la clave KA2 específicas de educación superior. En concreto, la relacionada con el abordaje de la transformación digital mediante el desarrollo de la preparación, la resiliencia y la capacidad digitales, con la estimulación de métodos de enseñanza innovadores y el soporte de las capacidades digitales en el sector de la educación superior. La propuesta es innovadora y no se basa en ninguna otra iniciativa anterior. También se valora positivamente que la propuesta es apropiada para crear sinergias entre diferentes ámbitos de la educación y la formación y entre universidades y hospitales. La propuesta es un claro ejemplo de un proyecto que potencialmente puede ser pertinente para otros ámbitos de la educación y la formación, por ejemplo, en otros ámbitos de la ciencia de la salud, además de en ámbitos más allá de la educación superior, involucrando a hospitales u otros centros de salud. La propuesta tiene un claro valor añadido dentro de la EU. Así, los resultados del proyecto se benefician si varios países, con diferentes sensibilidades comparten buenas prácticas y enfoques y además, los resultados no se lograrían con organizaciones de un solo país. **No se han identificado debilidades en este criterio.**

Criterio: Impacto
Puntuación: 24.0
Comentario:

Los resultados de la propuesta se integrarán de una manera directa en el desempeño de las funciones de las asociaciones implicadas, y del público objetivo en el que se identifican cinco actores: estudiantes de la rama sanitaria, tutores en las organizaciones sanitarias, tutores en las organizaciones educativas, gerentes de instituciones sanitarias y empresas relacionadas. La temática mejorará significativamente tanto la formación de profesores y alumnos, como su trabajo diario. Por lo tanto, se considera como una fortaleza que la propuesta que tenga impacto positivo sustancial en las organizaciones participantes, en todos sus ámbitos, académico, científico, de la salud y empresarial. El proyecto promueve la elaboración de Apps para móviles con el fin de que puedan usarse tanto en estudiantes de distintas especialidades de ciencias de la salud, como en hospitales, residencias. Los resultados son también relevantes para compañías y empresas que trabajen en el desarrollo de este tipo de aplicaciones. Por lo tanto, la transferibilidad de la propuesta está garantizada tanto dentro de las instituciones de los sectores de la educación superior (HEI) como en otros sectores. Además, los resultados son exportables a otros ámbitos fuera de las ciencias de la salud. Así, por la temática de la propuesta, por la calidad y organización de los resultados y por la pertinencia de las actividades de formación, se esperan beneficios transnacionales, interdisciplinares e intersectoriales. Además, la propuesta incluye medidas para garantizar la sostenibilidad y el impacto continuado una vez agotada la subvención, ya que se prevé el acceso gratuito a los productos intelectuales, con licencia del tipo creative commons. Otra de las fortalezas es que los dos eventos multiplicadores definidos en la propuesta son adecuados para alcanzar la difusión esperada de los Resultados del Proyecto. No obstante, se aprecian algunas debilidades en la solicitud de

subvención que podrían restar calidad a la misma, como son las relacionadas con la **falta de concreción en el uso de variables e indicadores para la evaluación del impacto sobre todo a nivel internacional. Además, el plan de difusión de los resultados apenas se detalla ni se concretan las actividades de este plan. Como aspectos de mejora se aprecia que la propuesta mejoraría si se indicara en la propuesta si la web del proyecto estará en servicio después de la finalización del proyecto. Además, es recomendable incluir los resultados del proyecto en las propias webs institucionales de los socios. Por último, además del uso de la Plataforma de Resultados de Proyectos Erasmus+, como herramienta de difusión puesta a disposición por la Comisión Europea se recomienda el uso de eTwinning o EPALE.**

Puntuación total: 82.0
Se alcanza el límite: YES
Comentarios para el solicitante:

El objetivo último de la propuesta, que es el desarrollo de aplicaciones para ser usadas por estudiantes de ciencias de la salud durante las prácticas, es relevante dentro de la prioridad de digitalización. Se presentan además técnicas de aprendizaje innovadoras, con aprendizaje basado en la práctica, la digitalización y la modernización. Otra de las fortalezas del proyecto es que se observa que el programa de trabajo se expone de forma clara y exhaustiva, identificando las diferentes fases del mismo y los socios implicados. La propuesta es consistente atendiendo a los objetivos que se plantea y las actividades que se incluyen para su consecución. La composición del equipo, que combina organizaciones del ámbito de la educación, investigación y empresas es adecuada. El reparto de tareas y responsabilidades entre los socios está lo suficientemente justificado y detallado. También se valora positivamente que los resultados 1 y el 2 son pertinentes y se han descrito de forma adecuada. Es una fortaleza que se incluyen medidas concretas y lógicas para integrar los resultados del proyecto en el trabajo habitual de los socios, así como a nivel local, nacional y europeo, ya que cualquier Universidad y hospital se beneficiará de éstos. Asimismo, la propuesta incluye medidas para garantizar la sostenibilidad y el impacto continuado una vez agotada la subvención, ya que los resultados ofrecen potencial para su uso fuera de las organizaciones involucradas después de su finalización y porque se prevé el acceso gratuito a los productos intelectuales, con licencia del tipo Creative Commons. Por último, los 2 eventos multiplicadores definidos en la propuesta son adecuados para alcanzar la difusión esperada. **Como debilidades, destaca la falta de un plan de comunicación detallado, o una planificación previa que garantice que el flujo de la información entre socios fluya adecuadamente. Asimismo, tampoco se ha detallado un plan de control de gasto con suficiente exhaustividad. En cuanto a los resultados, el resultado 3 se observa como una debilidad y se considera que se debe reenfocar para crear una guía de buenas prácticas. Además, la organización de las reuniones transnacionales no es adecuada, ya que incluye más de un miembro por organización, y supera las seis reuniones permitidas. Como aspectos de mejora se recomienda indicar en la propuesta si la web del proyecto estará en servicio después de la finalización del proyecto. Asimismo, es recomendable incluir los resultados del proyecto en las propias webs institucionales de los socios, así como en la Plataforma de Resultados del Programa Erasmus +, eTwinning o EPALE.Por otra parte, sería recomendable proporcionar un mayor detalle de las personas involucradas en el proyecto del socio UNIVERSITAET DUISBURG-ESSEN.Finalmente, se recomienda disminuir el número de reuniones presenciales a 4, y realizar las restantes de manera virtual como medida de sostenibilidad medioambiental.**
