# Supplementary material for: Connecting Actors With the Introduction of Mobile Technology in Health Care Practice Placements (4D Project): Protocol for a Mixed Methods Study
Source: JMIR Res Protoc. 2024 Feb 8;13:e53284. doi: 10.2196/53284 (PMC10884912; doi:10.2196/53284)
Supplement: Multimedia Appendix 3 [file resprot_v13i1e53284_app3.pdf]

CONVENIO DE SUBVENCIÓN relativo a un:
Proyecto con varios beneficiarios en el marco del programa ERASMUS+[1]

NÚMERO DE CONVENIO – 2021-1-ES01-KA220-HED-000027496

El presente Convenio (en lo sucesivo, «el Convenio») se celebra entre las partes que siguen:

de una parte,

la **Agencia Nacional** (denominada en lo sucesivo «la AN»)

Servicio Español para la Internacionalización de la Educación (SEPIE)
C/ General Oraa, número 55, 28006, Madrid
CIF: Q2801566G,

debidamente representada a efectos de la firma del presente Convenio por Alfonso Gentil Álvarez-Ossorio, Director del SEPIE, y actuando por delegación de la Comisión Europea, denominada, en lo sucesivo, «la Comisión»,

y

de otra parte,

el «**coordinador**»

FUNDACIO TECNOCAMPUS MATARO-MARESME
AVINGUDA ERNEST LLUCH 32 08302 MATARO
G62034111
1354

E10158035 .

debidamente representado a efectos de la firma del presente Convenio por

Josep Lluís Checa López ,

así como los demás beneficiarios que figuran en el anexo II, debidamente representados a efectos de la firma del presente Convenio por el coordinador, en virtud del (de los) mandato(s) incluido(s) en el anexo V.

---

[1] **Reglamento (UE) 2021/817 del Parlamento Europeo y del Consejo, de 20 de mayo de 2021, por el que se establece Erasmus+, el Programa de la Unión para la educación y la formación, la juventud y el deporte, y se deroga el Reglamento (UE) n.º 1288/2013**

Salvo que se especifique lo contrario, las referencias al «beneficiario» y a los «beneficiarios» incluyen al coordinador.

Las partes mencionadas anteriormente

HAN CONVENIDO LO SIGUIENTE:

Observar las Condiciones Particulares («las Condiciones Particulares»), así como los siguientes anexos:

Anexo I	Condiciones Generales (en lo sucesivo, «las Condiciones Generales»)
Anexo II	Descripción del proyecto, presupuesto estimativo del Proyecto, lista de los demás beneficiarios
Anexo III	Normas financieras y contractuales
Anexo IV	Tarifas aplicables
Anexo V	Mandato(s) otorgado(s) por el (los) otro(s) beneficiario(s) al coordinador

que forman parte integrante del Convenio.

Lo dispuesto en las Condiciones Particulares del presente Convenio prevalecerá sobre lo dispuesto en los anexos.

Lo dispuesto en el anexo I, «Condiciones Generales», prevalecerá sobre lo dispuesto en los demás anexos. Lo dispuesto en el anexo III prevalecerá sobre lo dispuesto en los anexos II y IV.

En el anexo II, la parte relativa al presupuesto estimativo prevalecerá sobre la parte correspondiente a la descripción del Proyecto.

CONDICIONES PARTICULARES

Índice

CLÁUSULA I.1 – OBJETO DEL CONVENIO 3

CLÁUSULA I.2 – ENTRADA EN VIGOR Y PERÍODO DE EJECUCIÓN DEL CONVENIO 3

CLÁUSULA I.3 – IMPORTE MÁXIMO Y FORMA DE LA SUBVENCIÓN 3

CLÁUSULA I.4 – DISPOSICIONES SOBRE LA PRESENTACIÓN DE INFORMES Y LOS PAGOS 3

I.4.1 Pagos que deberán efectuarse 4

I.4.2 Primer pago de prefinanciación 4

I.4.3 Informes intermedios y pagos de prefinanciación adicionales 4

I.4.4 Informe final y solicitud de pago del saldo 5

I.4.5 Pago del saldo 5

I.4.6 Notificación de las cantidades adeudadas 6

I.4.7 Pagos que la AN deberá abonar al coordinador e intereses de demora 6

I.4.8 Pagos que el coordinador deberá abonar a los demás beneficiarios 7

I.4.9 Moneda de pago 7

I.4.10 Divisa para las solicitudes de pago y conversión a euros 7

I.4.11 Idioma de las solicitudes de pago y de los informes 7

I.4.12 Fecha de pago 7

I.4.13 Gastos de transferencia correspondientes a los pagos 8

CLÁUSULA I.5 — CUENTA BANCARIA PARA LOS PAGOS 8

CLÁUSULA I.6 –RESPONSABLE DEL TRATAMIENTO DE LOS DATOS Y DATOS DE CONTACTO DE LAS PARTES 8

I.6.1 Responsable del tratamiento de los datos 8

I.6.2 Datos de contacto de la AN 9

I.6.3 Datos de contacto de los beneficiarios 9

CLÁUSULA I.7 – DISPOSICIÓN ADICIONAL RELATIVA A LA PRESENTACIÓN DE INFORMES SOBRE EL CUMPLIMIENTO DE LAS OBLIGACIONES DE PROTECCIÓN DE DATOS 9

CLÁUSULA I.8 – DISPOSICIÓN ADICIONAL RELATIVA A LA FACILITACIÓN DE INFORMACIÓN A LOS PARTICIPANTES SOBRE EL TRATAMIENTO DE SUS DATOS PERSONALES 9

CLÁUSULA I.9 – PROTECCIÓN Y SEGURIDAD DE LOS PARTICIPANTES 9

CLÁUSULA I.10 – DISPOSICIONES ADICIONALES SOBRE LOS DERECHOS PREEXISTENTES Y LA UTILIZACIÓN DE LOS RESULTADOS, INCLUIDOS LOS DERECHOS DE PROPIEDAD INTELECTUAL E INDUSTRIAL 10

CLÁUSULA I.11 – UTILIZACIÓN DE HERRAMIENTAS INFORMÁTICAS... 10

I.11.1 Herramienta de elaboración de informes y gestión de Erasmus+.................................. 10

I.11.2 Plataforma de Resultados de los Proyectos Erasmus+ 11

CLÁUSULA I.12 – DISPOSICIONES ADICIONALES RELATIVAS A LA SUBCONTRATACIÓN................ 11

CLÁUSULA I.13 – DISPOSICIÓN ADICIONAL RELATIVA A LA VISIBILIDAD DE LA FINANCIACIÓN DE LA UNIÓN 11

CLÁUSULA I.14 – APOYO PARA LOS PARTICIPANTES 11

CLÁUSULA I.15 – PRESTACIÓN DE APOYO A LA INCLUSIÓN PARA PARTICIPANTES 12

CLAÚSULA I.16 – APOYO LINGÜÍSTICO EN LÍNEA (OLS) 12

CLÁUSULA I.17 – DISPOSICIONES ESPECIALES SOBRE TRANSFERENCIAS PRESUPUESTARIAS....... 12

CLÁUSULA I.18 – DISPOSICIÓN ADICIONAL RELATIVA AL SEGUIMIENTO Y LA EVALUACIÓN........ 13

CLÁUSULA I.19 – DISPOSICIONES PARTICULARES RELATIVAS A LA RESPONSABILIDAD FINANCIERA EN MATERIA DE RECUPERACIONES.. 13

CLÁUSULA I.20 – BENEFICIARIOS DE PAÍSES ASOCIADOS... 13

CLÁUSULA I.21 – BENEFICIARIOS QUE SON ORGANIZACIONES INTERNACIONALES 13

CLÁUSULA I.22– EXCEPCIONES ESPECÍFICAS AL ANEXO I — CONDICIONES GENERALES.............. 14

CLÁUSULA I.23 – NORMAS FINANCIERAS Y CONTRACTUALES ADICIONALES APLICABLES ÚNICAMENTE A PROYECTOS EN LOS QUE SE ORGANICEN ACTIVIDADES VIRTUALES DEBIDO A LA COVID-19.. 17

I.2. Cálculo y documentos justificativos de las contribuciones por unidad.............................. 17

II.2. Cálculo de los costes reales.. 18

CLÁUSULA I.1 – OBJETO DEL CONVENIO

I.1.1 De conformidad con lo dispuesto en las Condiciones Particulares, las Condiciones Generales y los demás anexos del Convenio, la AN ha decidido subvencionar
el Proyecto titulado:
4D in the Digitalisation of Learning in Practice Placement
en el marco del programa Erasmus+, Acción Clave 2: Asociaciones para la cooperación, según se describe en el anexo II.

I.1.2 Al firmar el presente Convenio, los beneficiarios aceptan la subvención y se comprometen a ejecutar el Proyecto bajo su propia responsabilidad.

CLÁUSULA I.2 – ENTRADA EN VIGOR Y PERÍODO DE EJECUCIÓN DEL CONVENIO

I.2.1 El Convenio entrará en vigor en la fecha en que lo firme la Parte que lo haga en último lugar.

I.2.2 El Proyecto tendrá una duración de 36 meses, del 01/11/2021 al 31/10/2024 .

CLÁUSULA I.3 – IMPORTE MÁXIMO Y FORMA DE LA SUBVENCIÓN

I.3.1 La **subvención tendrá un importe máximo de** 377.840,00 EUR.

I.3.2 En relación con el presupuesto estimativo especificado en el anexo II y con los costes subvencionables y las normas financieras especificados en el anexo III, la subvención consiste en:

a) el reembolso de los costes subvencionables de la acción («el reembolso de los costes subvencionables») que:

- i) se hayan efectuado realmente
- ii) se hayan declarado sobre la base de los costes por unidad
- iii) sean un reembolso de los costes declarados con arreglo a un tanto alzado
- iv) sean un reembolso de los costes declarados con arreglo a un tipo fijo: no aplicable
- v) sean un reembolso de los costes declarados sobre la base de las prácticas de contabilidad de costes habituales del socio: no aplicable

b) una contribución por unidad: no aplicable
c) una contribución a tanto alzado: no aplicable
d) una contribución a tipo fijo: no aplicable
e) una financiación no vinculada a los costes: no aplicable

CLÁUSULA I.4 – DISPOSICIONES SOBRE LA PRESENTACIÓN DE INFORMES Y LOS PAGOS

Se aplicarán las siguientes disposiciones relativas a la presentación de informes y los pagos:

I.4.1 Pagos que deberán efectuarse

La AN deberá abonar los siguientes pagos al coordinador:

- un primer pago de prefinanciación;
- pagos intermedios, sobre la base de las solicitudes de pagos intermedios a que se refiere la cláusula I.4.3;
- un pago del saldo, sobre la base de la solicitud del pago del saldo a que se refiere la cláusula I.4.4.

I.4.2 Primer pago de prefinanciación

La finalidad de la prefinanciación es proporcionar fondos de tesorería a los beneficiarios. La prefinanciación continúa siendo propiedad de la AN hasta el pago del saldo.

La AN deberá abonar al coordinador el primer pago de prefinanciación en dos cuotas, de la manera que se detalla a continuación:

- dentro de los 30 días posteriores a la entrada en vigor del Convenio, una primera cuota correspondiente al 20 % del importe máximo de la subvención previsto en la cláusula I.3.1, excepto si se aplica la cláusula II.24.

- dentro de los 60 días posteriores a la entrega por parte del coordinador del primer informe intermedio al que hace referencia la cláusula I.4.3, la AN abonará una segunda cuota correspondiente al 20 % del importe máximo de la subvención previsto en la cláusula I.3.1., excepto si se aplica la cláusula II.24.

I.4.3 Informes intermedios y pagos de prefinanciación adicionales

A más tardar el 30 de junio 2022, el coordinador deberá completar un primer informe intermedio sobre la ejecución del Proyecto que abarque el ejercicio que va desde la fecha de inicio de la ejecución del Proyecto especificada en la cláusula I.2.2 hasta el 31 de mayo de 2022. Este informe se considerará como la solicitud de la segunda cuota del primer pago de prefinanciación.

A más tardar el 30 de junio de 2023, el coordinador deberá completar un segundo informe intermedio sobre la ejecución del Proyecto que abarque el ejercicio que va desde la fecha de inicio de la ejecución del Proyecto especificada en la cláusula I.2.2 hasta el 31 de mayo de 2023.

Siempre que este segundo informe intermedio demuestre que el coordinador utilizó al menos el 70 % del importe de los pagos de prefinanciación, dicho informe deberá considerarse como una solicitud de un pago de prefinanciación adicional y deberá precisar el importe que se solicita, el cual no deberá superar los 151.136,00 EUR, correspondiente al 40% del importe máximo total previsto en la cláusula I.3.1.

Si el informe intermedio demuestra que se utilizó menos del 70 % de los pagos de prefinanciación anteriores para cubrir costes del Proyecto, el coordinador deberá entregar un informe intermedio

adicional, una vez que se haya utilizado como mínimo el 70 % del importe de los pagos de prefinanciación, que deberá considerarse como una solicitud de un pago de prefinanciación adicional y deberá precisar el importe que se solicita, el cual no deberá superar los 151.136,00 EUR, correspondiente al 40% del importe máximo previsto en la cláusula I.3.1.

Sin perjuicio de lo dispuesto en las cláusulas II.24.1 y II.24.2 y una vez que la AN haya aprobado el informe, la AN deberá abonar al beneficiario el pago de prefinanciación adicional dentro de los 60 días naturales tras la recepción del informe intermedio.

Si el último informe intermedio demuestra que el beneficiario no podrá utilizar el importe máximo de la subvención previsto en la cláusula I.3.1 dentro del período contractual definido en la cláusula I.2.2, la AN hará una modificación mediante la cual se reducirá el importe máximo de la subvención según corresponda y, en el caso de que el importe máximo reducido de la subvención sea inferior al importe de prefinanciación transferido al beneficiario hasta esa fecha, la AN recuperará del beneficiario el importe en exceso de la prefinanciación, de conformidad con la cláusula II.26.

I.4.4 Informe final y solicitud de pago del saldo

Dentro de los 60 días naturales posteriores a la fecha de finalización del Proyecto prevista en la cláusula I.2.2, el coordinador deberá elaborar un informe final sobre la ejecución del Proyecto y, si procede, deberá cargar todos los resultados del Proyecto en la Plataforma de Resultados de los Proyectos Erasmus+, según se especifica en la cláusula I.11.2. El informe deberá contener la información necesaria para justificar el importe solicitado sobre la base de contribuciones por unidad, cuando la subvención consista en el reembolso de contribuciones por unidad, un importe a tanto alzado o los costes subvencionables en los que se haya incurrido realmente de conformidad con el anexo III.

El informe final se considerará como la solicitud por parte del coordinador del pago del saldo de la subvención.

El coordinador certificará que los datos proporcionados en la solicitud de pago del saldo sean completos, fidedignos y veraces. Certificará, asimismo, que los costes contraídos pueden considerarse subvencionables de conformidad con el Convenio y que la solicitud de pago está justificada por documentos adecuados que pueden presentarse en los controles o las auditorías descritos en la cláusula II.27.

I.4.5 Pago del saldo

El pago del saldo reembolsará o cubrirá la parte restante de los costes subvencionables contraídos por los beneficiarios para la ejecución del Proyecto.

La AN determinará el saldo adeudado deduciendo el importe total de los pagos de prefinanciación e intermedios, de haberlos, abonados del importe final de la subvención determinado con arreglo a lo dispuesto en la cláusula II.25.

Si el importe total de los pagos anteriores es superior al importe final de la subvención determinado con arreglo a lo dispuesto en la cláusula II.25, el pago del saldo adoptará la forma de recuperación con arreglo a lo dispuesto en la cláusula II.26.

Si el importe total de los pagos anteriores es inferior al importe final de la subvención determinado con arreglo a lo dispuesto en la cláusula II.25, la AN deberá abonar el saldo dentro de los 60 días naturales a partir de la fecha en que reciba los documentos contemplados en la cláusula I.4.4, a menos que sean aplicables las disposiciones de las cláusulas II.24.1 o II.24.2.

El pago estará condicionado a la aprobación de la solicitud de pago del saldo y de los documentos que la acompañen. Su aprobación no implicará el reconocimiento de la conformidad, autenticidad, integridad o exactitud de su contenido.

El importe que debe abonarse puede, no obstante, compensarse, sin consentimiento del coordinador, con cualquier otro importe adeudado por este a la AN, hasta el importe máximo indicado para el coordinador en cuestión, en el presupuesto estimativo en el Anexo II.

I.4.6 Notificación de las cantidades adeudadas

La AN deberá enviar una *notificación oficial* al coordinador en la que:

a) le informe sobre la cantidad adeudada; y
b) especifique si la notificación se refiere a un nuevo pago de prefinanciación o al pago del saldo.

Si se trata del pago del saldo, la AN deberá especificar asimismo el importe final de la subvención, determinado de conformidad con la cláusula II.25.

I.4.7 Pagos que la AN deberá abonar al coordinador e intereses de demora

La AN deberá realizar pagos al coordinador.

Si la AN no efectuara su pago dentro del plazo, el beneficiario tendrá derecho a percibir intereses de demora al tipo aplicado por el Banco Central Europeo para sus principales operaciones de refinanciación en euros («el tipo de referencia»), incrementado en tres puntos y medio. El tipo de referencia será el tipo vigente el primer día del mes en que expire el plazo de pago, tal como se publica en la serie C del *Diario Oficial de la Unión Europea*.

No se adeudarán intereses de demora cuando el beneficiario sea un Estado miembro de la Unión, incluidas las autoridades gubernamentales regionales y locales y otros organismos públicos que actúen en nombre y por cuenta del Estado miembro a efectos del Convenio.

Si la AN suspendiera el plazo de pago establecido en la cláusula II.24.2 o si suspendiera un pago efectivo según lo dispuesto en la cláusula II.24.1, estas acciones no podrán considerarse como casos de demora.

Los intereses de demora se calcularán sobre el periodo que va del día siguiente al del vencimiento del plazo de pago hasta, incluida, la fecha efectiva de pago tal como se establece en la cláusula I.4.12. La AN no tendrá en cuenta el interés pagadero para determinar el importe final de la subvención en el sentido de la cláusula II.25.

No obstante lo dispuesto en el párrafo primero, cuando los intereses calculados sean iguales o inferiores a 200 EUR, solo deberán pagarse al beneficiario previa solicitud de este presentada en el plazo de dos meses a partir de la recepción del pago demorado.

I.4.8 Pagos que el coordinador deberá abonar a los demás beneficiarios

El coordinador deberá abonar todos los pagos a los demás beneficiarios mediante transferencia bancaria y conservar los correspondientes justificantes de los importes transferidos a cada uno de los beneficiarios a efectos de control y auditoría, según lo dispuesto en la cláusula II.27.

I.4.9 Moneda de pago

La AN deberá efectuar sus pagos en euros.

I.4.10 Divisa para las solicitudes de pago y conversión a euros

Las solicitudes de pago deberán denominarse en euros.

Si la contabilidad general del beneficiario está denominada en una moneda distinta del euro, convertirá a euros los costes generados en otras monedas a la media de los tipos de cambio diarios publicados en la serie C del *Diario Oficial de la Unión Europea*, determinada a lo largo del ejercicio correspondiente (disponible en:
http://www.ecb.europa.eu/stats/exchange/eurofxref/html/index.en.html).

Si no se publicara en el *Diario Oficial de la Unión Europea* el tipo de cambio diario del euro para la moneda de que se trate, deberá convertirse utilizando el promedio de los tipos contables mensuales establecidos por la Comisión y publicados en su sitio web (http://ec.europa.eu/budget/contracts_grants/info_contracts/inforeuro/inforeuro_en.cfm), calculado a lo largo del ejercicio correspondiente.

El beneficiario cuya contabilidad general esté denominada en euros convertirá los costes generados en otras monedas en euros con arreglo a sus prácticas de contabilidad habituales.

I.4.11 Idioma de las solicitudes de pago y de los informes

Todas las solicitudes de pago y los informes deben presentarse en castellano o en inglés.

I.4.12 Fecha de pago

Se considera que la AN ha realizado los pagos en la fecha en que se carguen en la cuenta de la AN, a menos que el Derecho nacional lo disponga de otro modo.

I.4.13 Gastos de transferencia correspondientes a los pagos

Los gastos de transferencia correspondientes a los pagos se repartirán del siguiente modo:

a) la AN se hará cargo de los gastos de transferencia cargados por su banco;
b) el coordinador sufragará los gastos de transferencia cargados por su banco;
c) la Parte que ocasione la repetición de una transferencia soportará todos los gastos derivados de dicha repetición.

CLÁUSULA I.5 — CUENTA BANCARIA PARA LOS PAGOS

Todos los pagos deberán efectuarse en la cuenta bancaria del coordinador que se indica a continuación:

Nombre del banco: BANKIA

Denominación exacta del titular de la cuenta: FUNDACIO TECNOCAMPUS MATARO-MARESME

Número de cuenta completo (incluidos los códigos bancarios): ES14 2038 6697 7160 0009 4570

Código IBAN[2]: ES14 2038 6697 7160 0009 4570

CLÁUSULA I.6 –RESPONSABLE DEL TRATAMIENTO DE LOS DATOS Y DATOS DE CONTACTO DE LAS PARTES

I.6.1 Responsable del tratamiento de los datos

La entidad que actuará como responsable del tratamiento de los datos tal como establece la cláusula II.7 será:

Jefe de Unidad de B4
Dirección B – Juventud, Educación y Erasmus+
Dirección General de Educación, Juventud, Deporte y Cultura
Comisión Europea
B – 1049 BRUSELAS
Bélgica

[2] Código BIC o SWIFT para los países donde el código IBAN no sea aplicable.

I.6.2 Datos de contacto de la AN

Todas las comunicaciones dirigidas a la AN por el beneficiario deberán entregarse mediante el sistema electrónico de intercambio establecido por la AN en http://www.sepie.es/.

En ese caso, no serán aplicables la cláusula II.3.1, párrafo segundo, ni la cláusula II.3.2, párrafo segundo.

I.6.3 Datos de contacto de los beneficiarios

Todas las comunicaciones dirigidas por la AN al beneficiario deberán entregarse mediante el sistema electrónico de intercambio establecido por la AN en http://www.sepie.es/, dirigido a la siguiente dirección de correo electrónico: notificacions@tecnocampus.cat

En ese caso, no serán aplicables la cláusula II.3.1, párrafo segundo, ni la cláusula II.3.2, párrafo segundo.

CLÁUSULA I.7 – DISPOSICIÓN ADICIONAL RELATIVA A LA PRESENTACIÓN DE INFORMES SOBRE EL CUMPLIMIENTO DE LAS OBLIGACIONES DE PROTECCIÓN DE DATOS

En el informe final, los beneficiarios deberán informar sobre las medidas establecidas para garantizar el cumplimiento del Reglamento 2018/1725 en sus operaciones de tratamiento de datos, de conformidad con las obligaciones establecidas en la cláusula II.7, como mínimo, en relación con los siguientes temas: seguridad del tratamiento, confidencialidad del tratamiento, asistencia al responsable del tratamiento de los datos, retención de datos, contribución a auditorías, incluidas las inspecciones, establecimiento de registros de datos personales de todas las categorías de actividades de tratamiento realizadas en nombre del responsable del tratamiento.

CLÁUSULA I.8 – DISPOSICIÓN ADICIONAL RELATIVA A LA FACILITACIÓN DE INFORMACIÓN A LOS PARTICIPANTES SOBRE EL TRATAMIENTO DE SUS DATOS PERSONALES

Los beneficiarios facilitarán a los participantes la declaración de confidencialidad pertinente para el tratamiento de sus datos personales antes de que estos se codifiquen en los sistemas electrónicos para la gestión de las movilidades Erasmus+.

CLÁUSULA I.9 – PROTECCIÓN Y SEGURIDAD DE LOS PARTICIPANTES

Los beneficiarios establecerán procedimientos y mecanismos eficaces para garantizar la seguridad y la protección de los participantes en el Proyecto.

Los beneficiarios deberán garantizar que los participantes en las actividades de movilidad o en actividades de aprendizaje, enseñanza y formación cuenten con una cobertura de seguro.

Con anterioridad a la participación de menores en el Proyecto, los beneficiarios deberán garantizar el pleno respeto de la normativa aplicable sobre la protección y la seguridad de menores, tal como se define en la legislación vigente en los países de origen y de destino, incluidos, entre otros: el consentimiento parental o del tutor, los regímenes de seguro y los límites de edad.

CLÁUSULA I.10 – DISPOSICIONES ADICIONALES SOBRE LOS DERECHOS PREEXISTENTES Y LA UTILIZACIÓN DE LOS RESULTADOS, INCLUIDOS LOS DERECHOS DE PROPIEDAD INTELECTUAL E INDUSTRIAL

Además de lo dispuesto en la cláusula II.9.3, si los beneficiarios producen materiales educativos dentro del ámbito de aplicación del Proyecto, se deberá facilitar el acceso a dichos materiales a través de internet, sin cargo alguno y mediante licencia abierta[3].

Si los materiales o documentos están sujetos a derechos morales o derechos de terceros (incluidos los derechos de propiedad intelectual o los derechos de las personas físicas sobre su imagen y voz), los beneficiarios deberán asegurarse de cumplir con sus obligaciones en virtud de la cláusula II.9.2, en particular, mediante la obtención de la licencias y autorizaciones necesarias de los titulares de derechos de que se trate.

Los beneficiarios deberán asegurarse de que la dirección del sitio web utilizado sea válida y esté actualizada. Si se suspendiera el alojamiento del sitio web, los beneficiarios deberán eliminar el sitio web del sistema de registro de organizaciones para evitar el riesgo de que otra parte asuma el dominio y lo redirija a otros sitios web.

CLÁUSULA I.11 – UTILIZACIÓN DE HERRAMIENTAS INFORMÁTICAS

I.11.1 Herramienta de elaboración de informes y gestión de Erasmus+

El coordinador deberá utilizar la herramienta de elaboración de informes y gestión de Erasmus+ facilitada por la Comisión Europea para registrar toda la información relacionada con las actividades llevadas a cabo en el marco del Proyecto, incluidas las actividades que no contaron con el apoyo directo de una subvención de fondos de la UE, y para completar y presentar el informe de situación, el informe intermedio (si está disponible en la herramienta de elaboración de informes y gestión de Erasmus+ y para los casos especificados en la cláusula I.4.3) y el informe final.

[3] La licencia abierta es una manera de que el propietario de un trabajo dé permiso para que otras personas puedan usar el recurso. Cada recurso estará asociado a una licencia. Existen distintas licencias abiertas en función del nivel de permisos concedidos o las limitaciones impuestas, y el beneficiario tendrá la libertad de escoger la licencia específica que se aplicará a su trabajo. Cada recurso que se produzca deberá estar asociado a una licencia abierta. Una licencia abierta no es una cesión de los derechos de autor ni de los derechos de propiedad intelectual (DPI).

I.11.2 Plataforma de Resultados de los Proyectos Erasmus+

El coordinador presentará los resultados concretos del Proyecto en la Plataforma de Resultados de los Proyectos Erasmus+ (http://ec.europa.eu/programmes/erasmus-plus/projects/), de acuerdo con las instrucciones proporcionadas en dicha plataforma.

CLÁUSULA I.12 – DISPOSICIONES ADICIONALES RELATIVAS A LA SUBCONTRATACIÓN

Sin perjuicio de lo dispuesto en la cláusula II.11, los beneficiarios no deberán subcontratar ninguna de las actividades financiadas a través de la categoría presupuestaria «Resultados del proyecto».

Sin perjuicio de lo anterior, las disposiciones contenidas en la cláusula II.11.1, letras c) y d), no serán aplicables a ninguna de las categorías presupuestarias, a excepción de la de «Costes excepcionales».]

CLÁUSULA I.13 – DISPOSICIÓN ADICIONAL RELATIVA A LA VISIBILIDAD DE LA FINANCIACIÓN DE LA UNIÓN

Sin perjuicio de lo dispuesto en la cláusula II.8, los beneficiarios reconocerán el apoyo recibido en el marco del programa Erasmus+ en todo el material de comunicación y promoción, incluidos los sitios web y las redes sociales. Las directrices para los beneficiarios y otros terceros están disponibles en el sitio web http://sepie.es/comunicacion/imagen.html.

CLÁUSULA I.14 – APOYO PARA LOS PARTICIPANTES

Cuando la ejecución del Proyecto requiera que los beneficiarios concedan apoyo a los participantes, los beneficiarios concederán dicho apoyo de acuerdo con las condiciones especificadas en los anexos II y IV.

Los beneficiarios deberán:

- transferir el importe total de la ayuda financiera para viajes, apoyo individual, apoyo lingüístico a los participantes en actividades del proyecto, aplicando las tarifas de contribuciones por unidad especificadas en el anexo IV;

- o bien proporcionar la ayuda financiera para las mismas categorías presupuestarias a los participantes en actividades del proyecto en forma de provisión de los bienes y servicios necesarios. En ese caso, los beneficiarios deberán garantizar que los bienes y servicios cumplan con los requisitos exigidos de calidad y seguridad.

Los beneficiarios podrán combinar las dos opciones indicadas anteriormente, siempre que garanticen un trato justo y equitativo para todos los participantes. En ese caso, las condiciones aplicables a cada opción se aplicarán a las categorías presupuestarias a las que se aplique la opción correspondiente.

CLÁUSULA I.15 – PRESTACIÓN DE APOYO A LA INCLUSIÓN PARA PARTICIPANTES

En caso de que se hayan aprobado fondos para el apoyo a la inclusión para participantes, el beneficiario será responsable de garantizar que se proporcione la prefinanciación adecuada a los participantes con menos oportunidades que se beneficien de la subvención de inclusión. En particular, no podrá solicitarse a los participantes con menos oportunidades que prefinancien personalmente sus actividades.

CLAÚSULA I.16 – APOYO LINGÜÍSTICO EN LÍNEA (OLS)

No procede.

CLÁUSULA I.17 – DISPOSICIONES ESPECIALES SOBRE TRANSFERENCIAS PRESUPUESTARIAS

Se permitirá al beneficiario transferir fondos entre las distintas categorías presupuestarias, ocasionando un cambio del presupuesto estimativo y de las actividades relacionadas descritas en el anexo II, sin solicitar una modificación del Convenio, tal como se especifica en la cláusula II.13, a condición de que:

- el Proyecto se ejecute de conformidad con la solicitud relativa al proyecto aprobado y los objetivos globales descritos en el Anexo II

- y siempre que se respeten las normas específicas siguientes:

(a) **Resultados del proyecto, eventos multiplicadores y actividades de aprendizaje/enseñanza/formación:** El beneficiario podrá trasferir hasta el 30 % de los fondos asignados a cada una de estas categorías y asignarlos a cualquier otra categoría presupuestaria (respetando al mismo tiempo todos los demás límites definidos en la presente cláusula), sin necesidad de solicitar una modificación. El beneficiario podrá transferir fondos adicionales a estas categorías presupuestarias sin necesidad de solicitar una modificación.

(b) **Para la categoría presupuestaria «Actividades de aprendizaje/enseñanza/formación»:** El beneficiario podrá realizar cualquier transferencia entre los fondos asignados a «Actividades de aprendizaje/enseñanza/formación» sin necesidad de solicitar una modificación.

(c) **Reuniones transnacionales de proyecto y apoyo a la inclusión para organizaciones:** El beneficiario podrá trasferir hasta el 100 % de los fondos asignados y asignarlos a cualquier otra categoría presupuestaria (respetando al mismo tiempo todos los demás límites definidos en la presente cláusula). El beneficiario podrá transferir fondos adicionales a estas categorías presupuestarias sin necesidad de solicitar una modificación.

(d) **Gestión y ejecución del proyecto, y costes excepcionales relativos a la subcontratación y la adquisición de bienes y servicios:** El beneficiario podrá trasferir hasta el 100 % de los fondos

asignados a cada una de estas categorías y asignarlos a cualquier otra categoría presupuestaria (respetando al mismo tiempo todos los demás límites definidos en la presente cláusula). El beneficiario no podrá transferir fondos adicionales a estas categorías presupuestarias sin solicitar una modificación.

(e) **Apoyo a la inclusión para participantes:** El beneficiario podrá trasferir hasta el 15 % de los fondos asignados a esta categoría y asignarlos a cualesquiera otras categorías presupuestarias (respetando al mismo tiempo todos los demás límites definidos en la presente cláusula). El beneficiario podrá transferir fondos adicionales a esta categoría presupuestaria sin necesidad de solicitar una modificación.

(f) **Costes excepcionales para viajes de coste elevado y garantía financiera:** El beneficiario podrá trasferir hasta el 100 % de los fondos asignados y asignarlos a cualquier categoría presupuestaria (respetando al mismo tiempo todos los demás límites definidos en la presente cláusula). El beneficiario podrá transferir fondos adicionales a estas categorías presupuestarias sin necesidad de solicitar una modificación, y siempre y cuando se incluya una justificación pertinente de los gastos en el informe final.

No obstante lo dispuesto en las letras a) y b) de la presente cláusula, a efectos de establecer una garantía financiera, en la medida en que así lo requiera la AN en la cláusula I.4.2, los beneficiarios podrán transferir fondos asignados a cualquier categoría presupuestaria (distinta del apoyo a la inclusión para participantes) y asignarlos a la categoría presupuestaria «Costes excepcionales».

CLÁUSULA I.18 – DISPOSICIÓN ADICIONAL RELATIVA AL SEGUIMIENTO Y LA EVALUACIÓN

No procede.

CLÁUSULA I.19 – DISPOSICIONES PARTICULARES RELATIVAS A LA RESPONSABILIDAD FINANCIERA EN MATERIA DE RECUPERACIONES

La responsabilidad financiera de los beneficiarios distintos del coordinador se limitará al importe que reciba cada uno de ellos.

CLÁUSULA I.20 – BENEFICIARIOS DE PAÍSES ASOCIADOS

Las organizaciones de países asociados se comprometen a respetar los mismos principios que los beneficiarios de países del programa en lo que se refiere a la Carta Erasmus de Educación Superior, siempre que corresponda.

CLÁUSULA I.21 – BENEFICIARIOS QUE SON ORGANIZACIONES INTERNACIONALES

En el caso de que existieran beneficiarios con estatus jurídico de organismo internacional[4], la AN comprobará con la Comisión las disposiciones vigentes para cada tipo de organismo en relación con la financiación de la UE.

CLÁUSULA I.22– EXCEPCIONES ESPECÍFICAS AL ANEXO I — CONDICIONES GENERALES

1. A los efectos del presente Convenio, en el anexo I — Condiciones Generales, el término «la Comisión» significa «la AN», el término «acción» significa «proyecto» y el término «coste unitario» significa «contribución por unidad», salvo que se indique lo contrario.

A los efectos del presente Convenio, en el anexo I — Condiciones generales, el término «estados financieros» significa «la parte del informe dedicada al presupuesto», salvo que se indique lo contrario.

En las cláusulas II.4.1, II.7.1, II.8.2, II.27.1, II.27.3, la cláusula II.27.4, apartado primero, la cláusula II.27.8, apartado primero y la cláusula II.27.9, el término «la Comisión» debe entenderse como una referencia a «la AN y la Comisión».

En la cláusula II.12, el término «ayuda financiera» significa «ayuda» y el término «terceras partes» significa «participantes».

2. A los efectos del presente Convenio, no serán aplicables las cláusulas del anexo I, Condiciones Generales, que se indican a continuación: cláusula II.2.2, letra b), inciso ii); cláusula II.12.2; cláusula II.13.4; cláusula II.18.3 y cláusula II.27.7.

3. A los efectos del presente Convenio, los términos «*entidades afiliadas*», «*pago intermedio*» y «*tipo fijo*» no serán aplicables cuando aparezcan en las Condiciones Generales.

4. El epígrafe y la letra a) del párrafo primero de la cláusula II.9.3 deberán leerse como sigue:

> **«II.9.3 Derechos de uso de los resultados y de los derechos preexistentes por parte de la AN y la Unión**
>
> Los beneficiarios concederán a la AN y a la Unión los siguientes derechos de uso de los resultados del Proyecto:
>
> a) para sus propios fines, y en particular para ponerlos a disposición de personas que trabajen para la AN, las instituciones, las agencias y los organismos de la Unión y para las instituciones de los Estados miembros, así como para copiarlos y reproducirlos, total o parcialmente y en número ilimitado de ejemplares.».

[4] Las organizaciones internacionales son organismos internacionales del sector público creados mediante acuerdos intergubernamentales, así como agencias especializadas establecidas por dichas organizaciones u otras organizaciones sin ánimo de lucro asimiladas a organizaciones internacionales por decisión de la Comisión.

En el resto de esta cláusula, las referencias a la «Unión» se entenderán hechas a «la AN y/o la Unión».

5. La cláusula II.10.1, párrafo segundo, deberá leerse como sigue:

   «Los beneficiarios velarán por que la AN, la Comisión, el Tribunal de Cuentas Europeo y la Oficina Europea de Lucha contra el Fraude (OLAF) puedan ejercer sus derechos, en virtud de la cláusula II.27, también con respecto a los contratistas de los beneficiarios.».

6. Se agregará la letra l) a la cláusula II.17.3.1, que deberá leerse como sigue:

   «l) cuando todos los demás beneficiarios hayan denunciado que el coordinador no ejecuta el Proyecto según lo dispuesto en el anexo I o no cumple con otras obligaciones fundamentales impuestas por los términos del Convenio.».

7. La cláusula II.18 deberá leerse como sigue:

   «II.18.1 El Convenio se regirá por el siguiente régimen jurídico y por el orden que se indica:

   <u>Derecho comunitario</u>: Convocatoria de propuestas 2021- EAC/A01/2021 Programa Erasmus+ (2021/C103/11), las normas financieras aplicables al presupuesto general de la Unión y las dictadas en su desarrollo, así como los actos jurídicos contractuales que regulan las obligaciones entre la Agencia Nacional y la Comisión Europea, relativas a la ejecución del programa Erasmus+.

   <u>Derecho nacional</u>: Ley 39/2015, de 1 de octubre, del Procedimiento Administrativo Común de las Administraciones Públicas, Ley 40/2015, de 1 de octubre, de Régimen Jurídico del Sector Público, Ley 38/2003, de 17 de noviembre, General de Subvenciones, Ley 47/2003, de 26 de noviembre, General Presupuestaria, y su normativa de desarrollo, y en lo no previsto, serán de aplicación supletoria las restantes normas de Derecho administrativo general y especial, y en su defecto, el Derecho común, todo ello en cuanto no se contradigan con la normativa comunitaria.

   II.18.2 El órgano jurisdiccional competente determinado con arreglo al Derecho nacional aplicable será el único facultado para resolver cualquier conflicto entre la AN y el beneficiario en relación con la interpretación, la aplicación o la validez del presente Convenio, siempre que dicho conflicto no haya podido resolverse de forma amistosa.

   Podrán interponerse recursos contra los actos de la AN dentro de los plazos establecidos y ante los órganos correspondientes con arreglo a la Ley 39/2015, de 1 de octubre, del Procedimiento Administrativo Común de las Administraciones Públicas.».

8. En lo que respecta a la cláusula II.19.1, las condiciones de subvencionabilidad de los costes se complementan con el anexo III, secciones I.1 y II.1.

9. En lo que respecta a la cláusula II.20, las condiciones de identificabilidad y verificabilidad de los importes declarados se complementan con el anexo III, secciones I.2 y II.2.

10. La cláusula II.22, apartado primero, deberá leerse como sigue:

«Los beneficiarios podrán ajustar el presupuesto estimativo establecido en el anexo II mediante transferencias entre las distintas categorías presupuestarias, si el *Proyecto* se ejecuta según lo descrito en el anexo II. Este ajuste no exige una modificación del convenio, tal como se contempla en la cláusula II.13, siempre que se cumplan las condiciones establecidas en la cláusula I.17.».

11. La cláusula II.23, letra b), deberá leerse como sigue:

«b) siga sin presentar dicha solicitud en el plazo de otros 30 días naturales después de recibir un recordatorio por escrito enviado por la AN.».

12. La cláusula II.24.1.3, apartado primero, deberá leerse como sigue:

«Durante el período de suspensión de pagos, el coordinador no tendrá derecho a presentar las solicitudes de pago ni los documentos justificativos a los que se hace referencia en las cláusulas I.4.3 y I.4.4.».

13. En lo que respecta a la cláusula II.25.4, las condiciones para conceder una reducción debida a una ejecución incorrecta, una irregularidad, fraude o el incumplimiento de otras obligaciones se complementan con el anexo III, sección V.

14. La cláusula II.26.3, apartado tercero, deberá leerse como sigue:

«Si el pago no se hubiese realizado a más tardar en la fecha especificada en la nota de adeudo, la AN procederá a la recuperación del importe adeudado:

a) «[…] Podrá interponerse recurso contra dicha compensación ante el órgano jurisdiccional competente determinado en la cláusula II.18.2;

b) considerando a los beneficiarios conjunta y solidariamente responsables, hasta la contribución máxima indicada de la UE, para cada beneficiario, en el presupuesto estimativo (anexo II, con sus últimas modificaciones);

c) emprendiendo acciones legales con arreglo a lo dispuesto en la cláusula II.18.2 o en las Condiciones Particulares.».

15. La cláusula II.27.2, apartado tercero, deberá leerse como sigue:

«Los períodos indicados en los párrafos primero y segundo serán más prolongados si el Derecho nacional exige una duración mayor, o si están en marcha auditorías, recursos, litigios o reclamaciones relacionados con la subvención, incluidos los casos contemplados en la cláusula II.27.7. En esos casos, los beneficiarios deberán conservar los documentos hasta que se hayan cerrado esas auditorías, recursos, litigios o reclamaciones. ».

CLÁUSULA I.23 – NORMAS FINANCIERAS Y CONTRACTUALES ADICIONALES APLICABLES ÚNICAMENTE A PROYECTOS EN LOS QUE SE ORGANICEN ACTIVIDADES VIRTUALES DEBIDO A LA COVID-19

Las normas siguientes son adicionales a las ya incluidas en las cláusulas I.2 y II.2 del Anexo III, y se aplicarán únicamente en aquellos casos en que se hayan de organizar actividades virtuales debido a la COVID-19. La información sobre movilidades virtuales se proporcionará siguiendo las reglas establecidas en el convenio de subvención correspondiente.

Los beneficiarios podrán transferir hasta el 60% de los fondos asignados para las siguientes categorías presupuestarias: Reuniones transnacionales de proyecto, Eventos multiplicadores, Actividades de aprendizaje, enseñanza y formación y Costes excepcionales, a cualquier otra categoría presupuestaria en la que tenga lugar la actividad virtual con la excepción de Gestión y ejecución del proyecto y Costes excepcionales.

En lo que se refiere a Costes excepcionales, los beneficiarios podrán transferir hasta el 10% de los fondos asignados para cualquier categoría presupuestaria basada en contribuciones por unidad a la de Costes excepcionales para cubrir costes relacionados con la compra y/o alquiler de equipo y/o servicios necesarios para la realización de actividades de movilidad virtual debido a la COVID-19, incluso si no se hubieran adjudicado inicialmente fondos a esta categoría de costes excepcionales.

I.2. Cálculo y documentos justificativos de las contribuciones por unidad

Las condiciones estipuladas para la Gestión y ejecución del proyecto y eventos multiplicadores virtuales siguen siendo de aplicación.

A. Reuniones transnacionales de proyecto

No serán admisibles costes unitarios adicionales en caso de actividades virtuales. Los recursos disponibles en el presupuesto de "Gestión y ejecución del proyecto" cubrirán los costes relacionados con estas reuniones.

B. Actividades de aprendizaje, enseñanza y formación

a) Cálculo del importe de la subvención: el importe de la subvención adopta la forma de contribución por unidad en relación al apoyo individual.

- No se subvencionará el viaje.
- Podrá proporcionarse también apoyo lingüístico en el caso de actividades virtuales aplicando las mismas normas.

- Apoyo individual: el importe de la subvención se calcula multiplicando el número de días/meses de actividad virtual del participante por el 15% de la contribución por unidad aplicable por día/mes al tipo de participante y al país de destino de que se trate, como se especifica en el anexo IV del convenio. Los días de viaje

anteriores o posteriores a la actividad no podrán incluirse en el cálculo de la ayuda individual.

b) Hecho desencadenante:

- Apoyo individual: lo que condiciona el derecho a la subvención es que el participante haya realizado efectivamente la actividad.
- Apoyo lingüístico: lo que desencadena el derecho a la subvención es que el participante haya realizado una actividad de más de dos meses de duración y haya seguido efectivamente una preparación lingüística en la lengua de instrucción o de trabajo.

c) Documentos justificativos:

- Apoyo individual: Prueba de asistencia a la actividad en forma de declaración firmada por la organización de acogida en la que se especifiquen el nombre del participante y el propósito de la actividad, así como las fechas de inicio y conclusión de la actividad virtual.
- Apoyo lingüístico: Prueba de asistencia a cursos en forma de declaración firmada por el proveedor del curso en la que se especifiquen el nombre del participante, la lengua impartida y la duración del apoyo lingüístico.

II.2. Cálculo de los costes reales

A. Apoyo a la inclusión para participantes

El beneficiario podrá transferir fondos asignados para cualquier otra categoría presupuestaria a la de apoyo a la inclusión, incluso si no se hubieran adjudicado inicialmente fondos a esta categoría.

a) Cálculo del importe de la subvención: la subvención es un reembolso del 100 % de los costes subvencionables en los que realmente se ha incurrido.

b) Costes subvencionables: costes relacionados directamente con participantes con menos oportunidades para llevar a cabo actividades virtuales.

c) Documentos justificativos: facturas de los costes reales en las que se especifiquen el nombre y la dirección del organismo que expide la factura, el importe y la divisa, así como la fecha de la factura.

B. Costes excepcionales

a) Cálculo del importe de la subvención: la subvención es un reembolso del 75 % de los costes subvencionables en los que realmente se ha incurrido en la compra y/o alquiler de equipo y/o servicios.

b) Costes subvencionables: costes relativos a la compra y/o alquiler de equipo y/o servicios necesarios para la realización de actividades de movilidad virtual.

c) Documentos justificativos: prueba del pago de los costes incurridos sobre la base de facturas

en las que se especifiquen el nombre y la dirección del organismo que expide la factura, el importe y la divisa, así como la fecha de la factura.

FIRMAS

Por el beneficiario
Josep Lluís Checa López

Por la AN
Alfonso Gentil Álvarez-Ossorio

Hecho en Madrid.
